# Supplementary figures and images for: Yeast derlin Dfm1 employs a chaperone-like function to resolve misfolded membrane protein stress
Source: PLoS Biol. 2023 Jan 23;21(1):e3001950. doi: 10.1371/journal.pbio.3001950 (PMC9894555; doi:10.1371/journal.pbio.3001950)

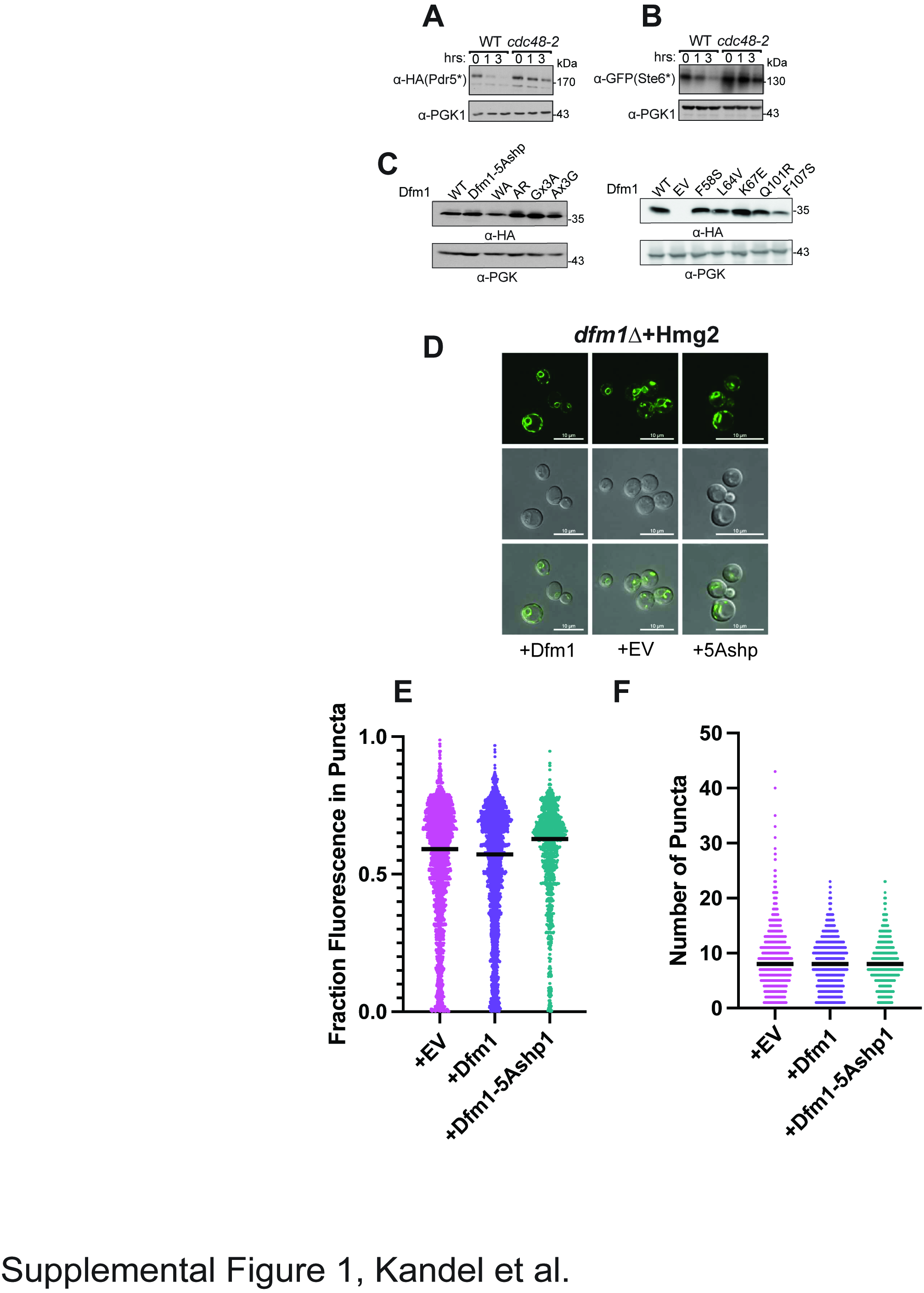

Supplement: S1 Fig — (A and B) WT and cdc48-2 strains were grown into log-phase at 30°C and degradation was measured by cycloheximide chase (CHX). After CHX addition, cells were lysed at the indicated times, and analyzed by SDS-PAGE and immunoblotted for Pdr5*-HA with α-HA and Ste6*-GFP with α-GFP. Three biological replicates (N = 3). (C) Steady-state levels of Dfm1 and corresponding Dfm1 mutants from dfm1Δ cells containing GALpr-HMG2-GFP that were used for growth assays in Fig 2. Cells were analyzed by SDS-PAGE and immunoblotted with α-HA. Three biological replicates (N = 3). (D) Representative confocal microscopy images of Hmg2-GFP in dfm1Δ cells with add-back of EV, WT DFM1, and DFM1-5Ashp. Five biological replicates were imaged, and 3 images were taken of each strain (N = 5). (E) Fraction of Hmg2-GFP in puncta for dfm1Δ cells with add-back of WT DFM1, EV, and DFM1-5Ashp. Each dot represents an individual cell. (F) Number of puncta per cell for dfm1Δ cells with add-back of WT DFM1, EV, and DFM1-5Ashp. Each dot represents an individual cell. Data information: The data underlying this figure can be found in Table M and N in S1 Data (Sheet 4). (TIF) [file pbio.3001950.s001.tif]

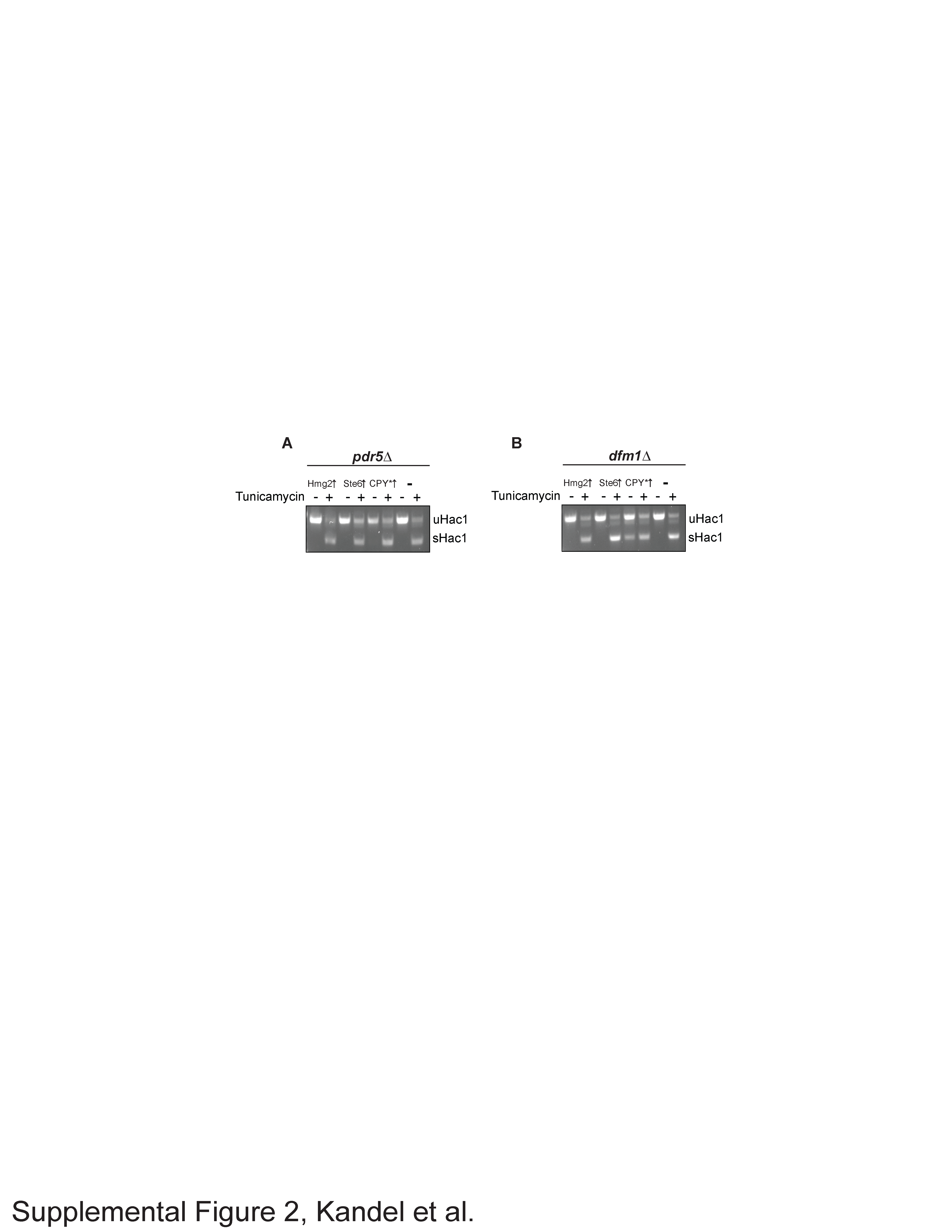

Supplement: S2 Fig — (A) pdr5Δ, pdr5Δdfm1Δ, and pdr5Δhrd1Δ cells containing either GALpr-HMG2-GFP or EV were compared for growth by dilution assay. Each strain was spotted 5-fold dilutions on glucose or galactose-containing plates to drive HMG2-GFP overexpression, and plates were incubated at 30°C. Three biological replicates and 2 technical replicates (N = 3). (B) Principal component 1 (PC1) and principal component 2 (PC2) values of each of the 2 biological replicates (N = 2) of RNA-seq samples for pdr5Δ, dfm1Δpdr5Δ, and hrd1Δpdr5Δ cells containing either GALpr-HMG2-GFP or EV. (C) PC1 and PC2 of sorted top 100 highest PC1 value genes from both replicates of dfm1Δpdr5Δ cells containing GALpr-HMG2-GFP. Red dots indicate Rpn4 target genes. Table indicates up-regulated genes that are targeted by Rpn4. (D) Top 10 GO terms and their enrichment factor for the set of 100 up-regulated genes with the highest PC1 scores. (E) Top 10 GO terms and their enrichment factor for the set of 100 down-regulated genes with the lowest PC1 scores. Data information: The data underlying this figure can be found in Table O–Q in S1 Data (Sheet 5) and Table R (Sheet 6). (TIFF) [file pbio.3001950.s002.tiff]

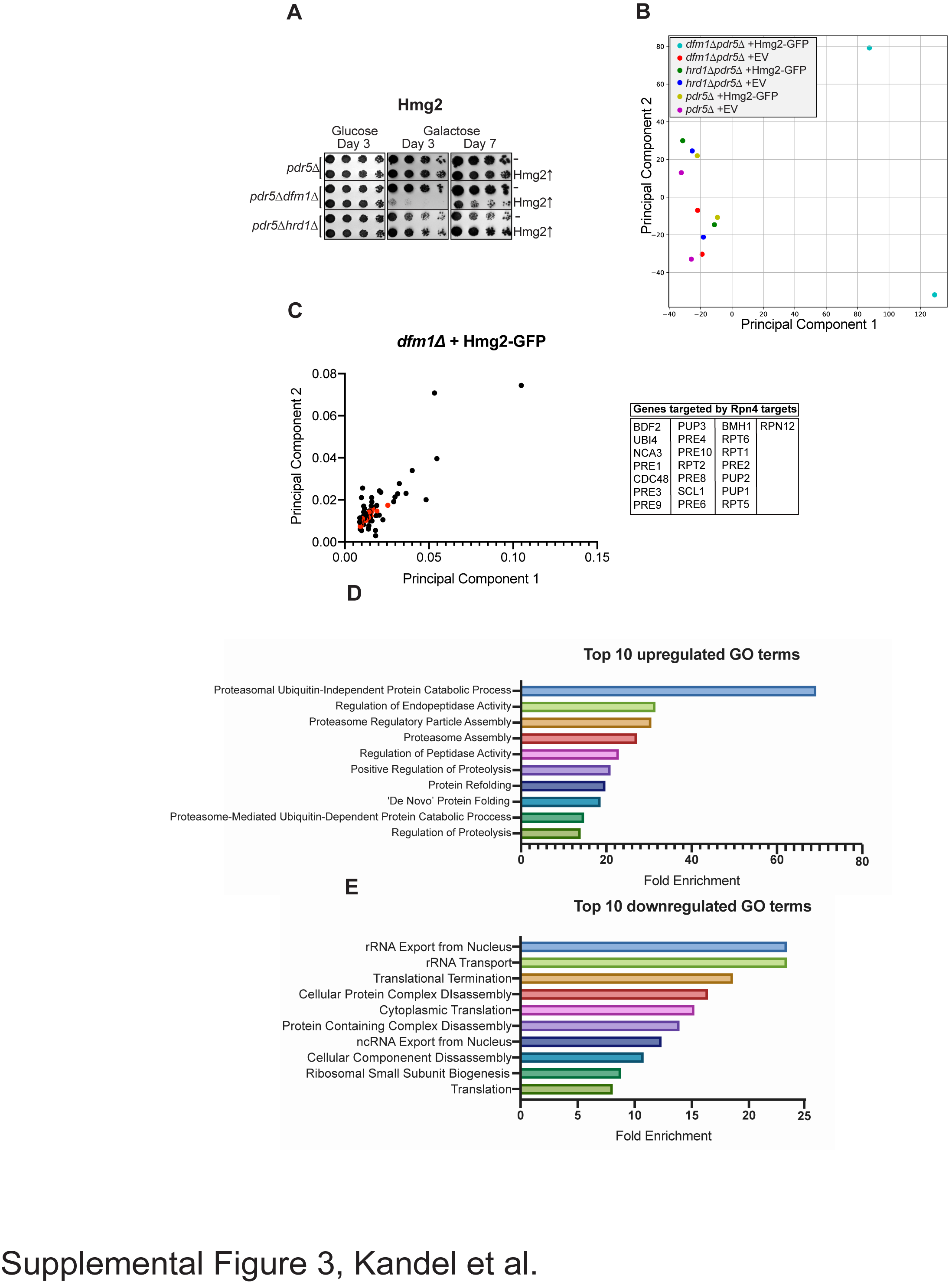

Supplement: S3 Fig — (A) PCR products of spliced and unspliced Hac1 transcripts. pdr5Δ cells containing GALpr-Hmg2-6MYC, GALpr-Ste6*-GFP, GALpr-CPY*-HA, or EV were treated with 0.2% galactose and 2 μg/mL tunicamycin (+) or an equivalent volume of DMSO (-). RNA was extracted from cells and cDNA was generated and used as a template for PCR. uHac1 represents unspliced Hac1 transcripts and sHac1 represents spliced Hac1. (B) Hac1 splicing assay as in (A) except using dfm1Δ cells. Data information: Images are representative of 3 biological replicates (N = 3). (TIF) [file pbio.3001950.s003.tif]

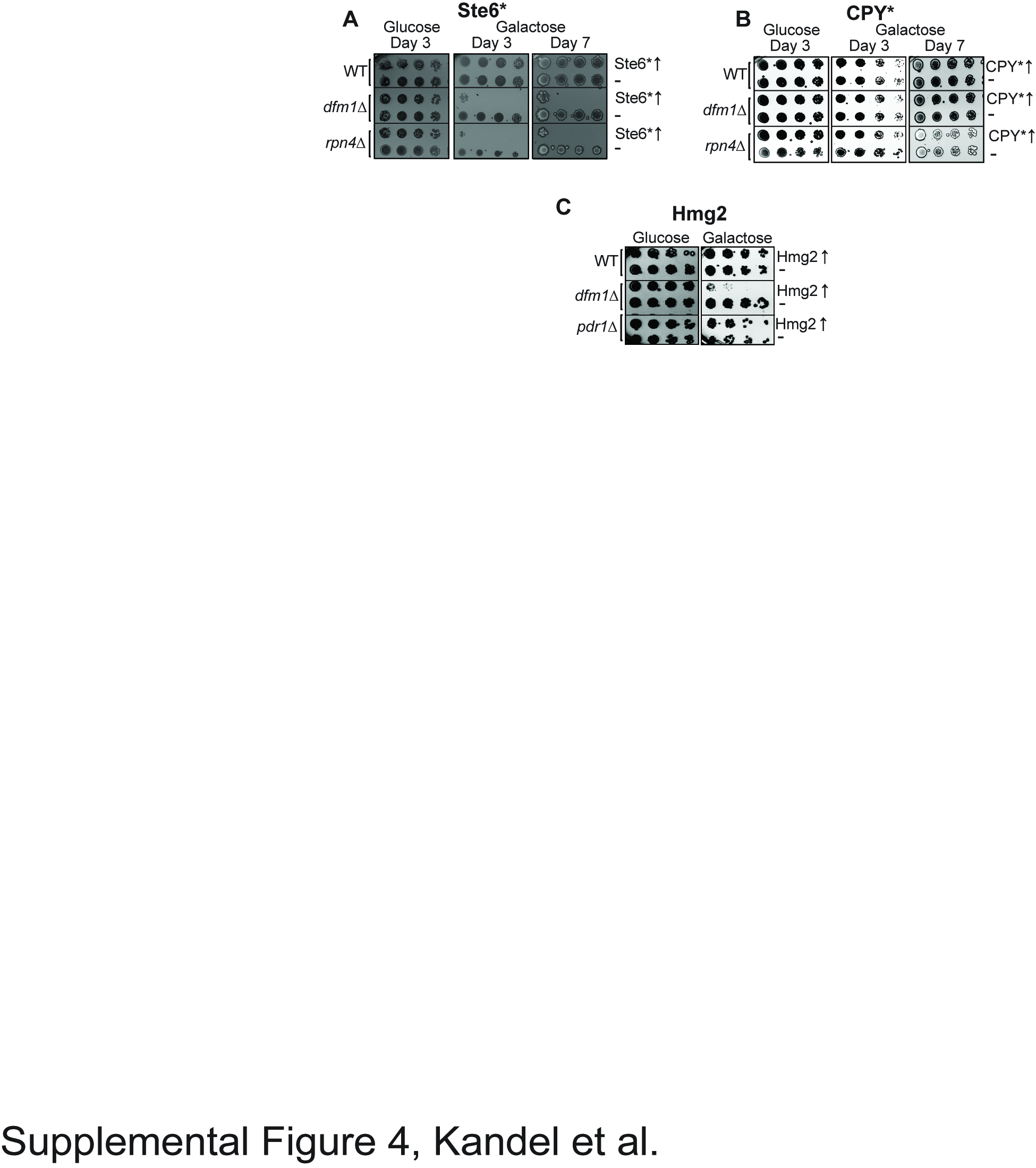

Supplement: S4 Fig — (A) WT, dfm1Δ, and rpn4Δ cells containing either GALpr-STE6*-GFP or EV were compared for growth by dilution assay. Each strain was spotted 5-fold dilutions on glucose or galactose-containing plates to drive Ste6*-GFP overexpression, and plates were incubated at 30°C. (B) Dilution assay as depicted in (A) except using cells containing GALpr-CPY*-HA or EV. (C) Dilution assay as described in (A) except using WT, dfm1Δ, and pdr1Δ cells containing either GALpr-HMG2-GFP or EV. Data information: All dilution growth assays were performed in 3 biological and 2 technical replicates (N = 3). (TIF) [file pbio.3001950.s004.tif]

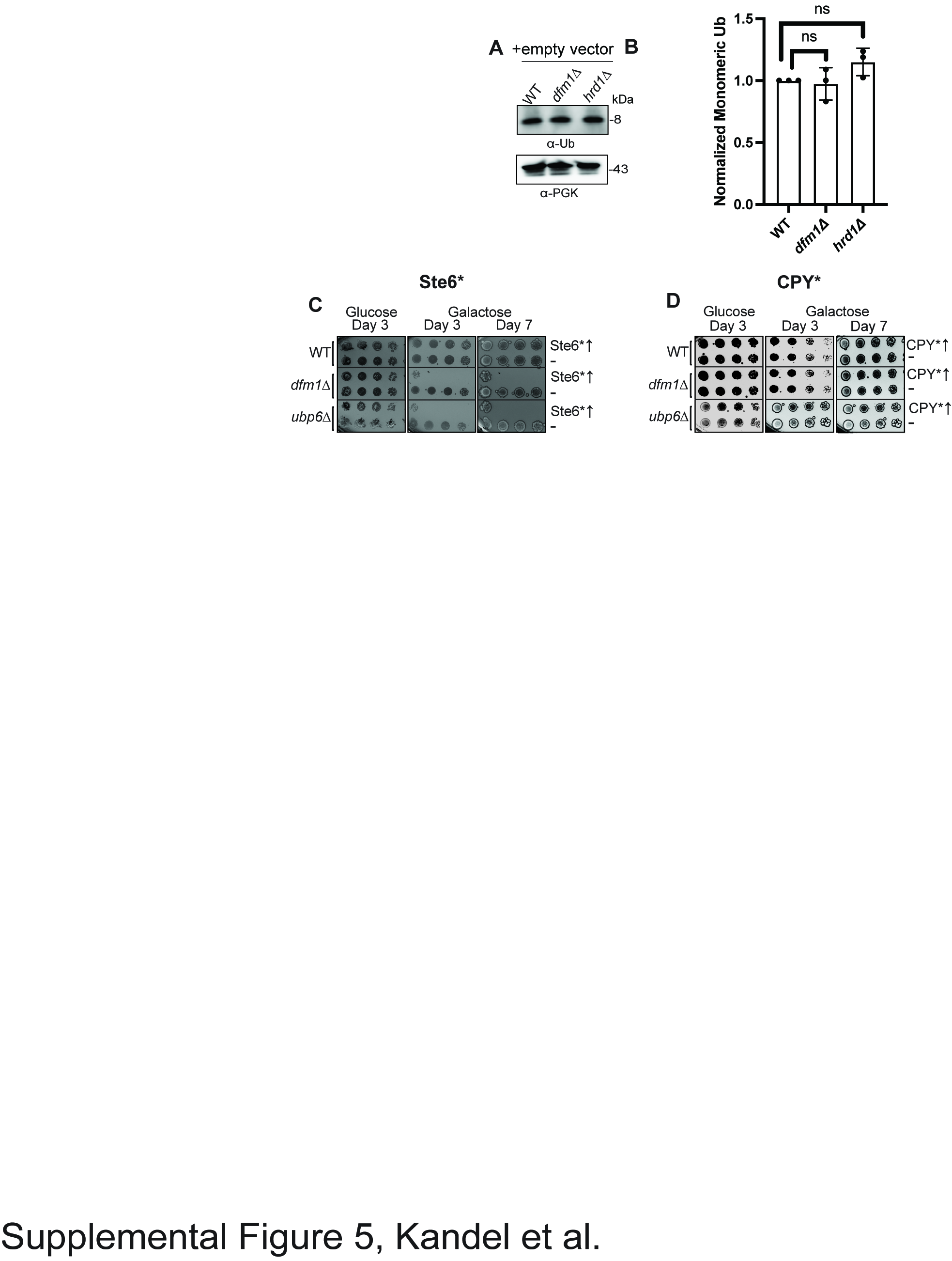

Supplement: S5 Fig — (A) Western blot of monomeric ubiquitin in WT, dfm1Δ, and hrd1Δ cells. Anti-ubiquitin was used to blot for ubiquitin and anti-PGK1 was used to blot for PGK1 as a loading control. (B) Quantification of western blots from (A). Each strain was normalized to PGK1 and the monomeric ubiquitin quantification of WT was used to normalize all strains. (C) WT, dfm1Δ, and ubp6Δ cells containing either GALpr-STE6*-GFP or EV were compared for growth by dilution assay. Each strain was spotted 5-fold dilutions on glucose or galactose-containing plates to drive Ste6*-GFP overexpression, and plates were incubated at 30°C. (D) Dilution assay as depicted in (C) except using cells containing GALpr-CPY*-HA or EV. Data information: All dilution growth assays were performed in 3 biological and 2 technical replicates (N = 3). For (B), all data are mean ± SEM, 3 biological replicates (N = 3); statistical significance is displayed as two-tailed unpaired t test, ns, not significant. The data underlying this figure can be found in Table S (Sheet 7). (TIF) [file pbio.3001950.s005.tif]

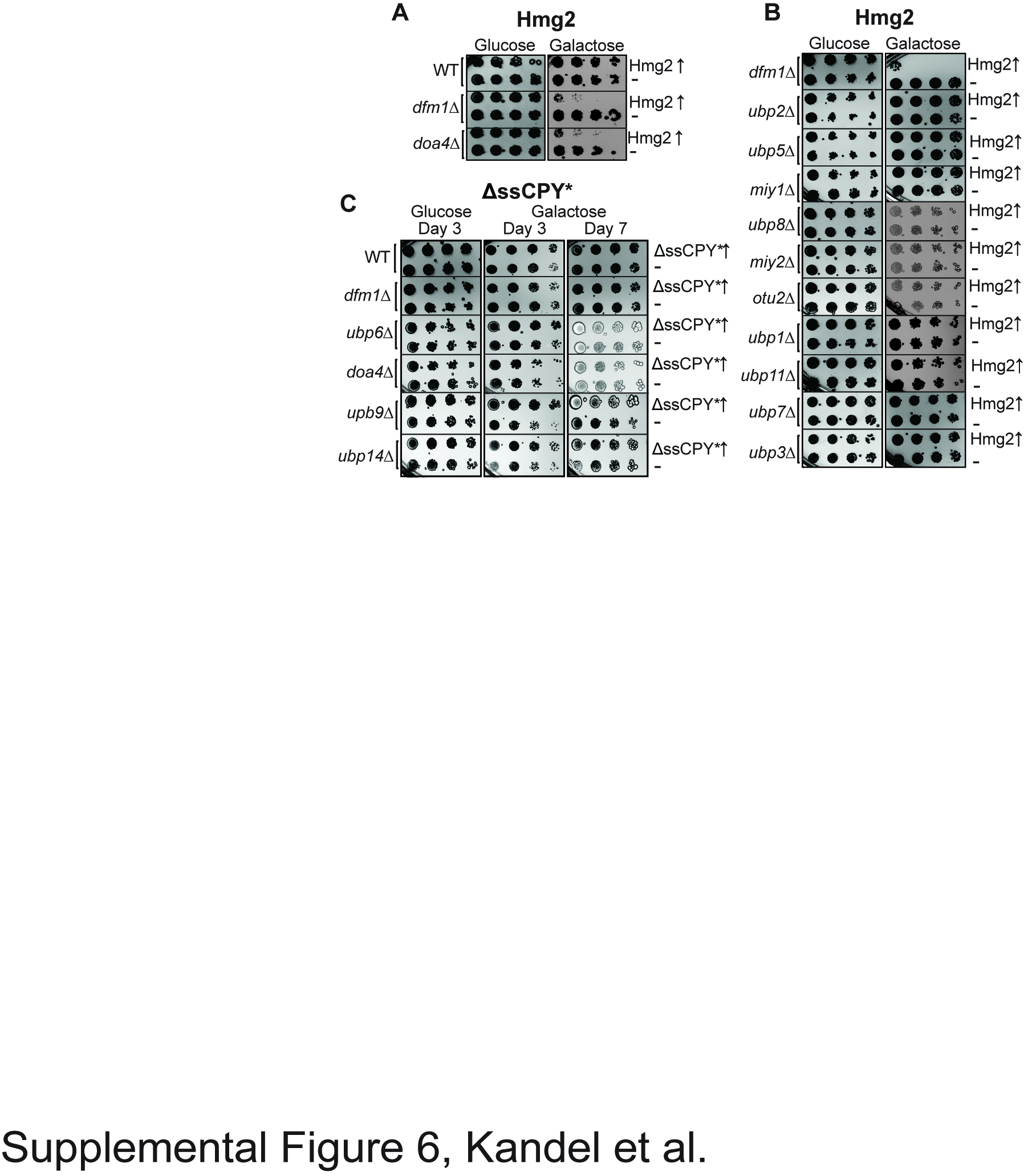

Supplement: S6 Fig — (A) WT, dfm1Δ, and doa4Δ cells containing either GALpr-HMG2-GFP or EV were compared for growth by dilution assay. Each strain was spotted 5-fold dilutions on glucose or galactose-containing plates to drive Hmg2-GFP overexpression, and plates were incubated at 30°C. (B) Dilution assay as in (A) except in dfm1Δ, ubp2Δ, ubp5Δ, miy1Δ, ubp8Δ, miy2Δ, otu2Δ, ubp1Δ, ubp11Δ, ubp7Δ, and ubp3Δ cells. (C) WT, dfm1Δ, ubp6Δ, doa4Δ, ubp9Δ, and ubp14Δ cells containing either GALpr-ΔssCPY*-Myc or EV were compared for growth by dilution assay. Each strain was spotted 5-fold dilutions on glucose or galactose-containing plates to drive Hmg2-GFP overexpression, and plates were incubated at 30°C. Data information: All dilution growth assays were performed in 3 biological and 2 technical replicates (N = 3). (TIF) [file pbio.3001950.s006.tif]

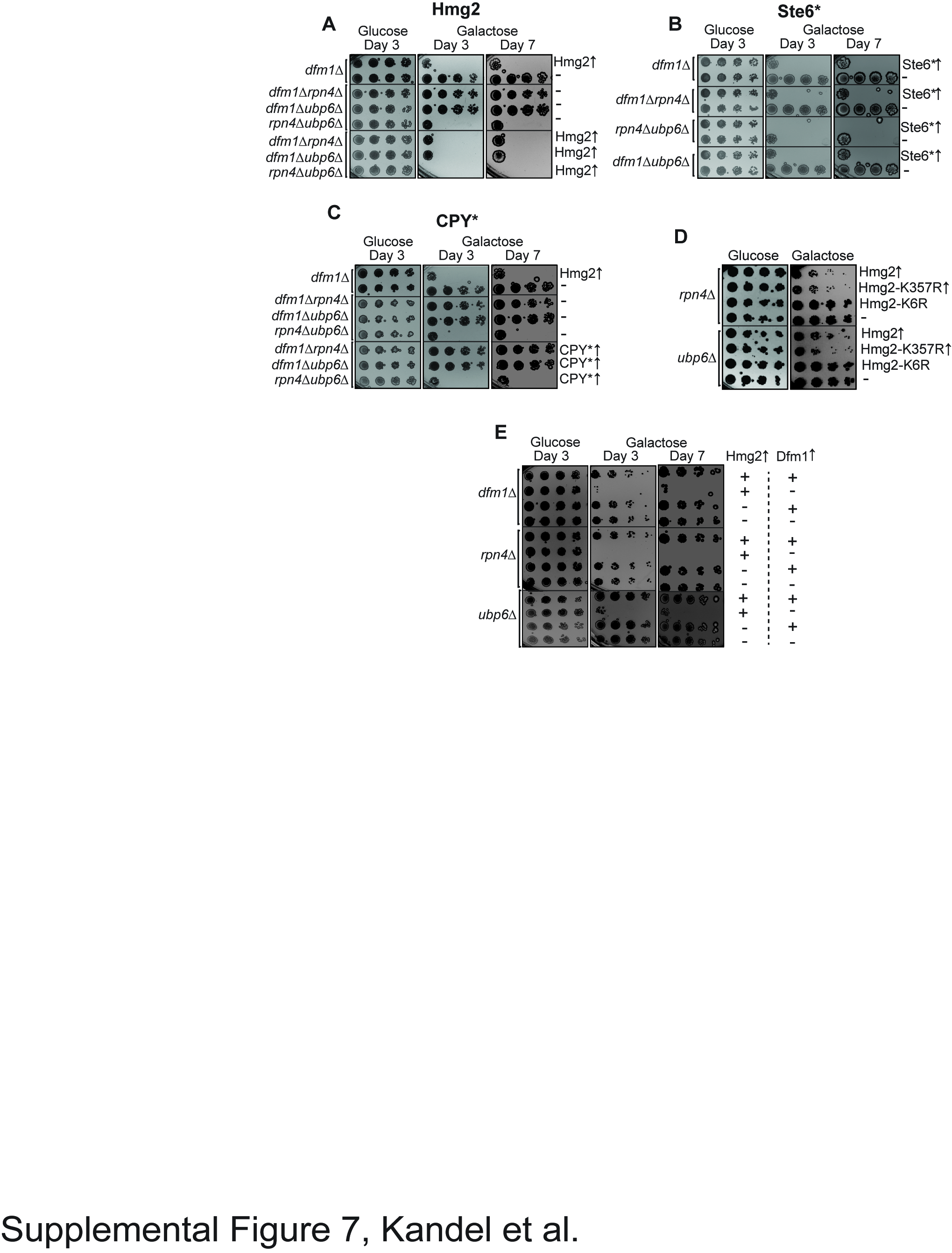

Supplement: S7 Fig — (A) dfm1Δ, dfm1Δrpn4Δ, dfm1Δubp6Δ, and rpn4Δubp6Δ cells containing either GALpr-HMG2-GFP or EV were compared for growth by dilution assay. Each strain was spotted 5-fold dilutions on glucose or galactose-containing plates to drive Hmg2-GFP overexpression, and plates were incubated at 30°C. (B) Dilution assays as depicted in (A) except using cells containing GALpr-STE6*-GFP. (C) Dilution assays as depicted in (A) except using cells containing GALpr-CPY*. (D) dfm1Δ, rpn4Δ, and ubp6Δ cells containing either GALpr-Hmg2-GFP or EV and GALpr-Dfm1-10xHis or EV were compared for growth by dilution assay. Each strain was spotted 5-fold dilutions on glucose or galactose-containing plates to drive Hmg2-GFP and Dfm1-10xHis overexpression, and plates were incubated at 30°C. (E) Dilution assay as described in (A) except using rpn4Δ and ubp6Δ cells containing either GALpr-Hmg2-GFP, GALpr-Hmg2 (K6R)-GFP, GALpr-Hmg2 (K357R)-GFP, GALpr-Hmg2 (K6R and K357R)-GFP, or EV. Data information: All dilution growth assays were performed in 3 biological and 2 technical replicates (N = 3). (TIF) [file pbio.3001950.s007.tif]
