# Supplementary material for: Yeast derlin Dfm1 employs a chaperone-like function to resolve misfolded membrane protein stress
Source: PLoS Biol. 2023 Jan 23;21(1):e3001950. doi: 10.1371/journal.pbio.3001950 (PMC9894555; doi:10.1371/journal.pbio.3001950)
Supplement: S1 Table — (DOCX) [file pbio.3001950.s008.docx]

**Table S1. Plasmids used in this study, Related to Figures 1-8**

| Plasmid | Gene |  |  |
| --- | --- | --- | --- |
| pRH1120  pRH3113  pRH3112  pRH3144  pRH317  pRH316  pRH1945 | YCp URA3 pGAL1-HMG2-GFP  YCp URA3 pGAL1-PDR5*-HA  YCp URA3 pGAL1-CPY*-HA  YCp URA3 pGAL1- Ste6-166p-3HA-GFP  YCp URA3  YCp LEU2  YIp ADE2 URA3 4xUPRE-GFP |  |  |
| pRH2890  pRH1997  pRH2812  pRH2813  pRH2826  pRH2827  pRH2013  pSN12  pSN11  pSN103  pSN104  pSN105  pSN59  pSN60  pSN93  pSN94  pSN95  pSN168  pSN86  pSN88  pSN100  pSN195  pSN196  pSN197  pSN199  pSN5  pSN193  pSN194  pSN39  pSN190  pRH613  pRH2312  pRH2058  pRH311  pRH1862  pRH469  pSN177  pRH2513  pSN189  pRH2880 | YCp LEU2 pDFM1-DFM1-3HA-5aShp  YCp LEU2 pDFM1-DFM1-3HA  YCp LEU2 pDFM1-DFM1-3HA-AxxxG  YCp LEU2 pDFM1-DFM1-3HA-GxxxA  YCp LEU2 pDFM1-DFM1-3HA-WA  YCp LEU2 pDFM1-DFM1-3HA-AR  YCp LEU2 pDFM1-DFM1-3HA  YIp ADE2 HIS3  YCp URA3  YIp ADE2 HIS3 pGAL1-HMG2-GFP-K357R  YIp ADE2 HIS3 pGAL1-HMG2-GFP-K6R  YIp ADE2 HIS3 pGAL1-HMG2-GFP  YCp LEU2 pDFM1-DFM1-3HA-F107S  YCp LEU2 pDFM1-DFM1-3HA-L64V  YCp LEU2 pDFM1-DFM1-3HA-K67E  YCp LEU2 pDFM1-DFM1-3HA-Q101R  YCp LEU2 pDFM1-DFM1-3HA-F57S  YCp ADE2 HIS3 pGAL1-HMG2-GFP-K6R-K357R  YIp ADE2 HIS3 pGAL1-STE6-166p-3HA-GFP  YIp ADE2 HIS3 pGAL1-HMG2-6MYC  YIp ADE2 HIS3 pGAL1-CPY*-HA  YCp URA3 CFTR-HA  YCp URA3 CFTR-HA-∆F508  YCp URA3 A1PiZ  YCp URA3 pGAL1-DFM1-6HIS  YCp URA3  YIp ADE2 LEU2 pADH1-Derlin-1-MYC  YIp ADE2 LEU2 pADH1-Derlin-2-MYC  YIp ADE2 LEU2  YCp URA3 pCUP1-HBT-Ubiquitin  YIp ADE2 pTDH3-HMG2-GFP  YCp HIS3 pPGK1-PDR5*-HA  YCp URA3 pPGK1-STE6-166p-3HA-GFP  YIp TRP1  YIp ADE2 URA3 pTDH3-HMG2-GFP-K6R  YIp URA3 HMG2-GFP  YCp URA3 CPY*-GFP  YIp TRP1 pHRD1-Hrd1-5MYC  YCp URA3 pGAL-∆ssCPY-MYC  YIp TRP1 pTDH3-SEC61-GFP |  |  |
|  |  |  |  |
|  |  |  |  |
|  |  |  |  |
|  |  |  |  |
|  |  |  |  |
|  |  |  |  |
|  |  |  |  |
