# Supplementary material for: Yeast derlin Dfm1 employs a chaperone-like function to resolve misfolded membrane protein stress
Source: PLoS Biol. 2023 Jan 23;21(1):e3001950. doi: 10.1371/journal.pbio.3001950 (PMC9894555; doi:10.1371/journal.pbio.3001950)
Supplement: S2 Table — (DOCX) [file pbio.3001950.s009.docx]

| **Table S2. Yeast strains used in this study** | | |  |
| --- | --- | --- | --- |
| **Strain** | **Genotype** | **Reference** | |
| RHY10520 | Mata *ADE2 met15∆0 LYS2(LYS+) ura3∆0 TRP1 leu2∆0 his3∆1 pdr5∆::KanMX*  *CEN::URA3* | This study | |
| RHY10519 | \| *Mata ADE2 met15∆0 LYS2(LYS+) ura3∆0 TRP1 leu2∆0 his3∆1 pdr5∆::KanMX* \| \| --- \|   *CEN::URA3::GAL1pr-HMG2-GFP* | This study | |
| RHY10518 | *Mata ADE2 met15∆0 LYS2(LYS+) ura3∆0 TRP1 leu2∆0 his3∆1 dfm1∆::KanMX*  *CEN::URA3* | This study | |
| RHY10517 | *Mata ADE2 met15∆0 LYS2(LYS+) ura3∆0 TRP1 leu2∆0 his3∆1*  *dfm1∆::KanMX*  *CEN::URA3::GAL1pr-HMG2-GFP* | This study | |
| RHY10655 | *Mata ADE2 met15∆0 LYS2(LYS+) ura3∆0 TRP1 leu2∆0 his3∆1hrd1∆::KanMX*  *CEN::URA3* | This study | |
| RHY10654 | \| *Mata ADE2 met15∆0 LYS2(LYS+) ura3∆0 TRP1 leu2∆0 his3∆1 hrd1∆::KanMX* \| \| --- \|   *CEN::URA3::GAL1pr-HMG2-GFP* | This study | |
| RHY11580 | *Mata ADE2 met15∆0 LYS2(LYS+) ura3∆0 TRP1 leu2∆0 his3∆1 pdr5∆::KanMX*  *CEN::URA3::GAL1pr-PDR5*-HA* | This study | |
| RHY11581 | *Mata ADE2 met15∆0 LYS2(LYS+) ura3∆0 TRP1 leu2∆0 his3∆1 dfm1∆::KanMX*  *CEN::URA3::GAL1pr-PDR5*-HA* | This study | |
| RHY11583 | *Mata ADE2 met15∆0 LYS2(LYS+) ura3∆0 TRP1 leu2∆0 his3∆1 hrd1∆::KanMX*  *CEN::URA3::GAL1pr-PDR5*-HA* | This study | |
| RHY11576 | *Mata ADE2 met15∆0 LYS2(LYS+) ura3∆0 TRP1 leu2∆0 his3∆1 pdr5∆::KanMX*  *CEN::URA3::GAL1pr-CPY*-HA* | This study | |
| RHY11577 | *Mata ADE2 met15∆0 LYS2(LYS+) ura3∆0 TRP1 leu2∆0 his3∆1 dfm1∆::KanMX*  *CEN::URA3::GAL1pr-CPY*-HA* | Neal et al., 2020 | |
| RHY11579 | *Mata ADE2 met15∆0 LYS2(LYS+) ura3∆0 TRP1 leu2∆0 his3∆1 hrd1∆::KanMX*  *CEN::URA3::GAL1pr-CPY*-HA* | Neal et al., 2020 | |
| \| RHY11817 \| \| --- \| \|  \| | \| *Mata ADE2 MET2 lys2-801 ura3∆0 TRP1 leu2∆0 his3∆200* \| \| --- \| \| *dfm1∆::KanMX hrd1∆::CloNAT*  *CEN::URA3* \| | Neal et al., 2020 | |
| RHY11818 | \| *Mata ADE2 MET2 lys2-801 ura3∆0 TRP1 leu2∆0 his3∆200* \| \| --- \| \| *dfm1∆::KanMX hrd1∆::CloNAT*  *CEN::URA3::GAL1pr-HMG2-GFP* \| | This study | |
| RHY 11867 | \| *Mata ADE2 met15∆0 LYS2(LYS+) ura3∆0 TRP1 leu2∆0 his3∆1 pdr5∆::KanMX* \| \| --- \| \| *CEN::URA3::GAL1pr-STE6-166p-3HA-GFP* \| | Neal et al., 2018 | |
| \| RHY 11868 \| \| --- \| \|  \| \|  \| \|  \| | \| *Mata ADE2 met15∆0 LYS2(LYS+) ura3∆0 TRP1 leu2∆0 his3∆1 dfm1∆::KanMX* \| \| --- \| \| *CEN::URA3::GAL1pr-STE6-166p-3HA-GFP* \| | Neal et al., 2018 | |
| RHY 11869 | \| *Mata ADE2 met15∆0 LYS2(LYS+) ura3∆0 TRP1 leu2∆0 his3∆1 hrd1∆::KanMX* \| \| --- \| \| *CEN::URA3::GAL1pr-STE6-166p-3HA-GFP* \| | Neal et al., 2020 | |
| RHY 11826 | \| *Mata ADE2 MET2 lys2-801 ura3∆0 TRP1 leu2∆0 his3∆200* \| \| --- \| \| *dfm1∆::KanMX hrd1∆::CloNAT*  *CEN::URA3::GAL1pr-STE6-166p-3HA-GFP* \| | This study | |
| RHY 11873 | \| *Mata ADE2 met15∆0 LYS2(LYS+) ura3-52 trp1::hisG leu2∆ his3∆1 dfm1∆::CloNAT doa10∆::HphMx* \| \| --- \| \| *CEN::URA3::GAL1pr-STE6-166p-3HA-GFP* \| | This study | |
| RHY 11908 | \| *Mata ADE2 met15∆0 LYS2(LYS+) ura3-52 trp1::hisG leu2∆ his3∆1 doa10∆::HphMx* \| \| --- \| \| *CEN::URA3::GAL1pr-STE6-166p-3HA-GFP* \| | This study | |
| RHY 11906 | \| *Mata ADE2 met15∆0 LYS2(LYS+) ura3-52 trp1::hisG leu2∆ his3∆1 doa10∆::HphMx* \| \| --- \| \| *CEN::URA3* \| | Neal et al., 2020 | |
| RHY 11907 | \| *Mata ADE2 met15∆0 LYS2(LYS+) ura3-52 trp1::hisG leu2∆ his3∆1 doa10∆::HphMx* \| \| --- \| \| *CEN::URA3::GAL1pr-HMG2-GFP* \| | Neal et al., 2020 | |
| RHY 11914 | \| *Mata ADE2 met15∆0 LYS2(LYS+) ura3-52 trp1::hisG leu2∆ his3∆1 dfm1∆::CloNAT doa10∆::HphMx* \| \| --- \| \| *CEN::URA3* \| | This study | |
| RHY 11915 | \| *Mata ADE2 met15∆0 LYS2(LYS+) ura3-52 trp1::hisG leu2∆ his3∆1 dfm1∆::CloNAT doa10∆::HphMx* \| \| --- \| \| *CEN::URA3::GAL1pr-HMG2-GFP* \| | This study | |
| \| RHY 11226 \| \| --- \| | \| *Mata ADE2 met15∆0 LYS2(LYS+) ura3∆0 TRP1 leu2∆0 his3∆1 dfm1∆::KanMX* \| \| --- \| \| *CEN::URA3 CEN::LEU2::pDFM1-DFM1-3HA* \| | Neal et al., 2018 | |
| RHY 11227 | \| *Mata ADE2 met15∆0 LYS2(LYS+) ura3∆0 TRP1 leu2∆0 his3∆1 dfm1∆::KanMX* \| \| --- \| \| *CEN::URA3:: GAL1pr-HMG2-GFP CEN::LEU2::pDFM1-DFM1-3HA* \| | Neal et al., 2018 | |
| \| RHY 11216 \| \| --- \| \|  \| | *Mata ADE2 met15∆0 LYS2(LYS+) ura3∆0 TRP1 leu2∆0 his3∆1 dfm1∆::KanMX*  *CEN::URA3 CEN::LEU2* | Neal et al., 2018 | |
| \| RHY11217 \| \| --- \| | \| *Mata ADE2 met15∆0 LYS2(LYS+) ura3∆0 TRP1 leu2∆0 his3∆1 dfm1∆::KanMX* \| \| --- \| \| *CEN::URA3:: GAL1pr-HMG2-GFP CEN::LEU2* \| | Neal et al., 2018 | |
| RHY11222 | \| *Mata ADE2 met15∆0 LYS2(LYS+) ura3∆0 TRP1 leu2∆0 his3∆1 dfm1∆::KanMX* \| \| --- \| \| *CEN::URA3 CEN::LEU2::pDFM1-DFM1-3HA-WA* \| | Neal et al., 2018 | |
| RHY11223 | \| *Mata ADE2 met15∆0 LYS2(LYS+) ura3∆0 TRP1 leu2∆0 his3∆1 dfm1∆::KanMX* \| \| --- \| \| *CEN::URA3:: GAL1pr-HMG2-GFP CEN::LEU2::pDFM1-DFM1-3HA-WA* \| | Neal et al., 2018 | |
| RHY11224 | \| *Mata ADE2 met15∆0 LYS2(LYS+) ura3∆0 TRP1 leu2∆0 his3∆1 dfm1∆::KanMX* \| \| --- \| \| *CEN::URA3 CEN::LEU2::pDFM1-DFM1-3HA-AR* \| | Neal et al., 2018 | |
| \| RHY11225 \| \| --- \| | \| *Mata ADE2 met15∆0 LYS2(LYS+) ura3∆0 TRP1 leu2∆0 his3∆1 dfm1∆::KanMX* \| \| --- \| \| *CEN::URA3:: GAL1pr-HMG2-GFP CEN::LEU2::pDFM1-DFM1-3HA-AR* \| | Neal et al., 2018 | |
| RHY11218 | \| *Mata ADE2 met15∆0 LYS2(LYS+) ura3∆0 TRP1 leu2∆0 his3∆1 dfm1∆::KanMX* \| \| --- \| \| *CEN::URA3 CEN::LEU2::pDFM1-DFM1-3HA-AxxxG* \| | Neal et al., 2018 | |
| RHY11219 | \| *Mata ADE2 met15∆0 LYS2(LYS+) ura3∆0 TRP1 leu2∆0 his3∆1 dfm1∆::KanMX* \| \| --- \| \| *CEN::URA3:: GAL1pr-HMG2-GFP CEN::LEU2::pDFM1-DFM1-3HA-AxxxG* \| | Neal et al., 2018 | |
| RHY11220 | \| *Mata ADE2 met15∆0 LYS2(LYS+) ura3∆0 TRP1 leu2∆0 his3∆1 dfm1∆::KanMX* \| \| --- \| \| *CEN::URA3 CEN::LEU2::pDFM1-DFM1-3HA-GxxxA* \| | Neal et al., 2018 | |
| RHY11221 | \| *Mata ADE2 met15∆0 LYS2(LYS+) ura3∆0 TRP1 leu2∆0 his3∆1 dfm1∆::KanMX* \| \| --- \| \| *CEN::URA3:: GAL1pr-HMG2-GFP CEN::LEU2::pDFM1-DFM1-3HA-GxxxA* \| | Neal et al., 2018 | |
| RHY11073 | \| *Mata ADE2 met15∆0 LYS2(LYS+) ura3∆0 TRP1 leu2∆0 his3∆1 dfm1∆::KanMX* \| \| --- \| \| *CEN::URA3 CEN::LEU2::pDFM1-DFM1-3HA-5aShp* \| | Neal et al., 2018 | |
| RHY11074 | \| *Mata ADE2 met15∆0 LYS2(LYS+) ura3∆0 TRP1 leu2∆0 his3∆1 dfm1∆::KanMX* \| \| --- \| \| *CEN::URA3:: GAL1pr-HMG2-GFP CEN::LEU2::pDFM1-DFM1-3HA-5aShp* \| | Neal et al., 2018 | |
| SEN141 | Mata *ade2::ADE2::HIS3::pGAL1::Hmg2-GFP met2 lys2-801 ura3-52 trp1::hisG leu2∆ his3∆200* | This study | |
| SEN142 | Mata *ade2::ADE2::HIS3 met2 lys2-801 ura3-5, trp1::hisG leu2∆ his3∆200* | This study | |
| SEN149 | Mata *ade2::ADE2::HIS3 met2 lys2-801 ura3-52 trp1::hisG leu2∆ his3∆200 dfm1∆::KanMX* | This study | |
| SEN165 | Mata *ade2::ADE2::HIS3::pGAL1::Hmg2-GFP met2 lys2-801 ura3-52, trp1::hisG leu2∆ his3∆200 dfm1∆::KanMX* | This study | |
| SEN407 | Mata *ade2-101 met2 lys2-801 ura3-5, trp1::hisG leu2∆ his3∆200*  *CEN::ADE2::HIS3::pGAL1-Hmg2-GFP-K6R-K357R* | This study | |
| SEN408 | Mata *ade2-101 met2 lys2-801 ura3-5, trp1::hisG leu2∆ his3∆200 dfm1∆::KanMX*  *CEN::ADE2::HIS3::pGAL1-Hmg2-GFP-K6R-K357R* | This study | |
| SEN139 | Mata *ade2::ADE2::HIS3::pGAL1::Hmg2-GFP-K357R met2 lys2-801 ura3-52 trp1::hisG leu2∆ his3∆200* | This study | |
| SEN147 | Mata *ade2::ADE2::HIS3::pGAL1::Hmg2-GFP-K357R met2 lys2-801 ura3-52 trp1::hisG leu2∆ his3∆200 dfm1∆::KanMX* | This study | |
| SEN140 | Mata *ade2::ADE2::HIS3::pGAL1::Hmg2-GFP-K6R met2 lys2-801 ura3-52 trp1::hisG leu2∆ his3∆200* | This study | |
| SEN148 | Mata *ade2::ADE2::HIS3::pGAL1::Hmg2-GFP-K6R met2 lys2-801 ura3-52 trp1::hisG leu2∆ his3∆200 dfm1∆::KanMX* | This study | |
| SEN182 | Mata *ade2::ADE2::HIS3 met2 lys2-801 ura3-52 trp1::hisG leu2∆ his3∆200 dfm1∆::KanMX*  *CEN::LEU2::pDFM1-DFM1-3HA* | This study | |
| SEN183 | Mata *ade2::ADE2::HIS3 met2 lys2-801 ura3-52 trp1::hisG leu2∆ his3∆200 dfm1∆::KanMX*  *CEN::LEU2* | This study | |
| SEN192 | Mata *ade2::ADE2::HIS3::pGAL1::Hmg2-GFP met2 lys2-801 ura3-52, trp1::hisG leu2∆ his3∆200 dfm1∆::KanMX*  *CEN::LEU2::pDFM1-DFM1-3HA* | This study | |
| SEN193 | Mata *ade2::ADE2::HIS3::pGAL1::Hmg2-GFP met2 lys2-801 ura3-52, trp1::hisG leu2∆ his3∆200 dfm1∆::KanMX*  *CEN::LEU2* | This study | |
| SEN250 | Mata *ade2::ADE2::HIS3 met2 lys2-801 ura3-52 trp1::hisG leu2∆ his3∆200 dfm1∆::KanMX*  *CEN::LEU2::pDFM1-DFM1-3HA-F107S* | This study | |
| SEN251 | Mata *ade2::ADE2::HIS3 met2 lys2-801 ura3-52 trp1::hisG leu2∆ his3∆200 dfm1∆::KanMX*  *CEN::LEU2::pDFM1-DFM1-3HA-L64V* | This study | |
| SEN252 | Mata *ade2::ADE2::HIS3 met2 lys2-801 ura3-52 trp1::hisG leu2∆ his3∆200 dfm1∆::KanMX*  *CEN::LEU2::pDFM1-DFM1-3HA-K67E* | This study | |
| SEN253 | Mata *ade2::ADE2::HIS3 met2 lys2-801 ura3-52 trp1::hisG leu2∆ his3∆200 dfm1∆::KanMX*  *CEN::LEU2::pDFM1-DFM1-3HA-Q101R* | This study | |
| SEN254 | Mata *ade2::ADE2::HIS3 met2 lys2-801 ura3-52 trp1::hisG leu2∆ his3∆200 dfm1∆::KanMX*  *CEN::LEU2::pDFM1-DFM1-3HA-F58S* | This study | |
| SEN256 | Mata *ade2::ADE2::HIS3::pGAL1::Hmg2-GFP met2 lys2-801 ura3-52, trp1::hisG leu2∆ his3∆200 dfm1∆::KanMX*  *CEN::LEU2::pDFM1-DFM1-3HA-F107S* | This study | |
| SEN257 | Mata *ade2::ADE2::HIS3::pGAL1::Hmg2-GFP met2 lys2-801 ura3-52, trp1::hisG leu2∆ his3∆200 dfm1∆::KanMX*  *CEN::LEU2::pDFM1-DFM1-3HA-L64V* | This study | |
| SEN258 | Mata *ade2::ADE2::HIS3::pGAL1::Hmg2-GFP met2 lys2-801 ura3-52, trp1::hisG leu2∆ his3∆200 dfm1∆::KanMX*  *CEN::LEU2::pDFM1-DFM1-3HA-K67E* | This study | |
| SEN259 | Mata *ade2::ADE2::HIS3::pGAL1::Hmg2-GFP met2 lys2-801 ura3-52, trp1::hisG leu2∆ his3∆200 dfm1∆::KanMX*  *CEN::LEU2::pDFM1-DFM1-3HA-Q101R* | This study | |
| SEN260 | Mata *ade2::ADE2::HIS3::pGAL1::Hmg2-GFP met2 lys2-801 ura3-52, trp1::hisG leu2∆ his3∆200 dfm1∆::KanMX*  *CEN::LEU2::pDFM1-DFM1-3HA-F58S* | This study | |
| SEN103 | Mata *ade2::ADE2::URA3::4xUPRE-GFP met15∆0 LYS2 (LYS+) ura3∆0 TRP1 leu2∆0 his3∆1 pdr5∆::KanMX*  *pGAL1::CPY*-HA* | This study | |
| SEN111 | Mata *ade2::ADE2::URA3::4xUPRE-GFP met15∆0 LYS2 (LYS+) ura3∆0 TRP1 leu2∆0 his3∆1 dfm1∆:: KanMX*  *pGAL1::CPY*-HA* | This study | |
| SEN73 | Mata *ade2::ADE2::URA3::4xUPRE-GFP met15∆0 LYS2 (LYS+) ura3∆0 TRP1 leu2∆0 his3∆1 pdr5∆::KanMX*  *pGAL1::STE6-166p-3HA-GFP* | This study | |
| SEN75 | Mata *ade2::ADE2::URA3::4xUPRE-GFP met15∆0 LYS2 (LYS+) ura3∆0 TRP1 leu2∆0 his3∆1 dfm1∆:: KanMX*  *pGAL1::STE6-166p-3HA-GFP* | This study | |
| SEN76 | Mata *ade2::ADE2::URA3::4xUPRE-GFP met15∆0 LYS2 (LYS+) ura3∆0 TRP1 leu2∆0 his3∆1 ADE2::HIS3 pdr5∆::KanMX* | This study | |
| SEN68 | Mata *ade2::ADE2::URA3::4xUPRE-GFP met15∆0 LYS2 (LYS+) ura3∆0 TRP1 leu2∆0 his3∆1 ADE2::HIS3 dfm1∆::KanMX* | This study | |
| SEN70 | Mata *ade2::ADE2::URA3::4xUPRE-GFP met15∆0 LYS2 (LYS+) ura3∆0 TRP1 leu2∆0 his3∆1 dfm1∆::KanMX pGAL1::HMG2-6MYC* | This study | |
| SEN71 | Mata *ade2::ADE2::URA3::4xUPRE-GFP met15∆0 LYS2 (LYS+) ura3∆0 TRP1 leu2∆0 his3∆1 ADE2::HIS3 dfm1∆::KanMX* | This study | |
| SEN155 | Mata *ADE2::HIS3 met15∆0 LYS2 (LYS+) ura3∆0 TRP1 leu2∆0 his3∆1 rpn4∆::KanMX* | This study | |
| SEN166 | Mata *ADE2::HIS3::pGAL::Hmg2-GFP met15∆0 LYS2 (LYS+) ura3∆0 TRP1 leu2∆0 his3∆1 rpn4∆::KanMX* | This study | |
| SEN196 | Mata *ADE2::HIS3 met15∆0 LYS2 (LYS+) ura3∆0 TRP1 leu2∆0 his3∆1 ubp6∆::KanMX* | This study | |
| SEN197 | Mata *ADE2::HIS3::pGAL1::Hmg2-GFP met15∆0 LYS2 (LYS+) ura3∆0 TRP1 leu2∆0 his3∆1 ubp6∆::KanMX* | This study | |
| SEN411 | Mata *ade2::ADE2::HIS3::pGAL1::STE6-166-3HA-GFP met2 lys2-801 ura3-52 trp1::hisG leu2∆ his3∆200* | This study | |
| SEN412 | Mata *ADE2::HIS3::pGAL1::STE6-166p-3HA-GFP met15∆0 LYS2 (LYS+) ura3∆0 TRP1 leu2∆0 his3∆1 rpn4∆::KanMX* | This study | |
| SEN413 | Mata *ade2::ADE2::HIS3:: pGAL1::STE6-166p-3HA-GFP met2 lys2-801 ura3-52 trp1::hisG leu2∆ his3∆200 dfm1∆::KanMX* | This study | |
| SEN414 | Mata *ADE2::HIS3::pGAL1::STE6-166p-3HA-GFP met15∆0 LYS2 (LYS+) ura3∆0 TRP1 leu2∆0 his3∆1 ubp6∆::KanMX* | This study | |
| SEN269 | Mata *ADE2::HIS3::pGAL1::CPY*-HA met15∆0 LYS2 (LYS+) ura3∆0 TRP1 leu2∆0 his3∆1 ubp6∆::KanMX* | This study | |
| SEN415 | Mata *ade2::ADE2::HIS3::pGAL1::CPY*-HA met2 lys2-801 ura3-52 trp1::hisG leu2∆ his3∆200* | This study | |
| SEN416 | Mata *ADE2::HIS3::pGAL1::CPY*-HA met15∆0 LYS2 (LYS+) ura3∆0 TRP1 leu2∆0 his3∆1 rpn4∆::KanMX* | This study | |
| SEN417 | Mata *ade2::ADE2::HIS3:: pGAL1::CPY*-HA met2 lys2-801 ura3-52 trp1::hisG leu2∆ his3∆200 dfm1∆::KanMX* | This study | |
| SEN270 | Mata *ADE2::HIS3::pGAL1::CPY*-HA met15∆0 LYS2 (LYS+) ura3∆0 TRP1 leu2∆0 his3∆1 rpn4∆::KanMX dfm1∆::CloNAT* | This study | |
| SEN271 | Mata *ADE2::HIS3::pGAL1::CPY*-HA met15∆0 LYS2 (LYS+) ura3∆0 TRP1 leu2∆0 his3∆1 rpn4∆::KanMX ubp6∆::CloNAT* | This study | |
| SEN272 | Mata *ADE2::HIS3::pGAL1::CPY*-HA met15∆0 LYS2 (LYS+) ura3∆0 TRP1 leu2∆0 his3∆1 ubp6∆::KanMX dfm1∆::CloNAT* | This study | |
| SEN499 | Mata *ADE2::HIS3::pGAL1::STE6-166p-3HA-GFP met15∆0 LYS2 (LYS+) ura3∆0 TRP1 leu2∆0 his3∆1 rpn4∆::KanMX dfm1∆::CloNAT* | This study | |
| SEN500 | Mata *ADE2::HIS3::pGAL1::STE6-166p-3HA-GFP met15∆0 LYS2 (LYS+) ura3∆0 TRP1 leu2∆0 his3∆1 rpn4∆::KanMX ubp6∆::CloNAT* | This study | |
| SEN501 | Mata *ADE2::HIS3::pGAL1::STE6-166p-3HA-GFP met15∆0 LYS2 (LYS+) ura3∆0 TRP1 leu2∆0 his3∆1 ubp6∆::KanMX dfm1∆::CloNAT* | This study | |
| SEN273 | Mata *ADE2::HIS3::pGAL1::HMG2-GFP met15∆0 LYS2 (LYS+) ura3∆0 TRP1 leu2∆0 his3∆1 rpn4∆::KanMX dfm1∆::CloNAT* | This study | |
| SEN276 | Mata *ADE2::HIS3 met15∆0 LYS2 (LYS+) ura3∆0 TRP1 leu2∆0 his3∆1 rpn4∆::KanMX dfm1∆::CloNAT* | This study | |
| SEN274 | Mata *ADE2::HIS3::pGAL1::HMG2-GFP met15∆0 LYS2 (LYS+) ura3∆0 TRP1 leu2∆0 his3∆1 rpn4∆::KanMX ubp6∆::CloNAT* | This study | |
| SEN277 | Mata *ADE2::HIS3 met15∆0 LYS2 (LYS+) ura3∆0 TRP1 leu2∆0 his3∆1 rpn4∆::KanMX ubp6∆::CloNAT* | This study | |
| SEN275 | Mata *ADE2::HIS3::pGAL1::HMG2-GFP met15∆0 LYS2 (LYS+) ura3∆0 TRP1 leu2∆0 his3∆1 ubp6∆::KanMX dfm1∆::CloNAT* | This study | |
| SEN278 | Mata *ADE2::HIS3 met15∆0 LYS2 (LYS+) ura3∆0 TRP1 leu2∆0 his3∆1 ubp6∆::KanMX dfm1∆::CloNAT* | This study | |
| SEN487 | Mata *ade2::ADE2::HIS3::pGAL1::Hmg2-GFP met2 lys2-801 ura3-52, trp1::hisG leu2∆ his3∆200 dfm1∆::KanMX*  *CEN::URA3::pGAL1::DFM1-6HIS* | This study | |
| SEN488 | Mata *ade2::ADE2::HIS3::pGAL1::Hmg2-GFP met2 lys2-801 ura3-52, trp1::hisG leu2∆ his3∆200 dfm1∆::KanMX*  *CEN::URA3* | This study | |
| SEN489 | Mata *ade2::ADE2::HIS3 met2 lys2-801 ura3-52, trp1::hisG leu2∆ his3∆200 dfm1∆::KanMX*  *CEN::URA3::pGAL1::DFM1-6HIS* | This study | |
| SEN490 | Mata *ade2::ADE2::HIS3 met2 lys2-801 ura3-52, trp1::hisG leu2∆ his3∆200 dfm1∆::KanMX*  *CEN::URA3* | This study | |
| SEN491 | Mata *ADE2::HIS3::pGAL::Hmg2-GFP met15∆0 LYS2 (LYS+) ura3∆0 TRP1 leu2∆0 his3∆1 rpn4∆::KanMX*  *CEN::URA3::pGAL1::DFM1-6HIS* | This study | |
| SEN492 | Mata *ADE2::HIS3::pGAL::Hmg2-GFP met15∆0 LYS2 (LYS+) ura3∆0 TRP1 leu2∆0 his3∆1 rpn4∆::KanMX*  *CEN::URA3* | This study | |
| SEN493 | Mata *ADE2::HIS3 met15∆0 LYS2 (LYS+) ura3∆0 TRP1 leu2∆0 his3∆1 rpn4∆::KanMX*  *CEN::URA3::pGAL1::DFM1-6HIS* | This study | |
| SEN494 | Mata *ADE2::HIS3 met15∆0 LYS2 (LYS+) ura3∆0 TRP1 leu2∆0 his3∆1 rpn4∆::KanMX*  *CEN::URA3* | This study | |
| SEN517 | Mata *ADE2::HIS3::pGAL::Hmg2-GFP met15∆0 LYS2 (LYS+) ura3∆0 TRP1 leu2∆0 his3∆1 ubp6∆::KanMX*  *CEN::URA3::pGAL1::DFM1-6HIS* | This study | |
| SEN518 | Mata *ADE2::HIS3::pGAL::Hmg2-GFP met15∆0 LYS2 (LYS+) ura3∆0 TRP1 leu2∆0 his3∆1 ubp6∆::KanMX*  *CEN::URA3* | This study | |
| SEN519 | Mata *ADE2::HIS3 met15∆0 LYS2 (LYS+) ura3∆0 TRP1 leu2∆0 his3∆1 ubp6∆::KanMX*  *CEN::URA3::pGAL1::DFM1-6HIS* | This study | |
| SEN520 | Mata *ADE2::HIS3 met15∆0 LYS2 (LYS+) ura3∆0 TRP1 leu2∆0 his3∆1 ubp6∆::KanMX*  *CEN::URA3* | This study | |
| SEN249 | Mata *ade2-101 met2 lys2-801 ura3-52 trp1::hisG::TRP1:: pTDH3-Hmg1p-MYC-Hrd1p-3HA-GFP leu2∆ his3∆200 hrd1∆::KanMX dfm1∆::CloNAT pdr5∆::HIS3* | This study | |
| SEN378 | Mata *ade2-101 met2 lys2-801 ura3-52 trp1::hisG::TRP1 leu2∆ his3∆200 hrd1∆::KanMX pdr5∆::HIS3* | This study | |
| SEN229 | Mata *ade2-101 met2 lys2-801 ura3-52 trp1::hisG::TRP1:: pTDH3-Hmg1p-MYC-Hrd1p-3HA-GFP leu2∆ his3∆200 hrd1∆::KanMX pdr5∆::HIS3* | This study | |
| SEN228 | Mata *ade2-101 met2 lys2-801 ura3-52 trp1::hisG::TRP1:: pTDH3-Hmg1p-MYC-Hrd1p-3HA-GFP leu2∆ his3∆200 pdr5∆::HIS3* | This study | |
| SEN377 | Mata *ade2-101 met2 lys2-801 ura3-52 trp1::hisG::TRP1 leu2∆ his3∆200 pdr5∆::HIS3* | This study | |
| SEN379 | Mata *ade2-101 met2 lys2-801 ura3-52 trp1::hisG::TRP1 leu2∆ his3∆200 hrd1∆::KanMX dfm1∆::CloNAT pdr5∆::HIS3* | This study | |
| SEN401 | Mata *ADE2::HIS3::pGAL1::Hmg2-GFP met15∆0 LYS2 (LYS+) ura3∆0 TRP1 leu2∆0 his3∆1 ubp9∆::KanMX* | This study | |
| SEN424 | Mata *ADE2::HIS3 met15∆0 LYS2 (LYS+) ura3∆0 TRP1 leu2∆0 his3∆1 ubp9∆::KanMX* | This study | |
| SEN446 | Mata *ADE2 met15∆0 LYS2 (LYS+) ura3∆0 TRP1 leu2∆0 his3∆1 ubp9∆::KanMX*  *CEN::URA3:: ∆ssCPY*-MYC* | This study | |
| SEN459 | Mata *ADE2 met15∆0 LYS2 (LYS+) ura3∆0 TRP1 leu2∆0 his3∆1 ubp14∆::KanMX*  *CEN::URA3* | This study | |
| SEN460 | Mata *ADE2 met15∆0 LYS2 (LYS+) ura3∆0 TRP1 leu2∆0 his3∆1 doa4∆::KanMX*  *CEN::URA3* | This study | |
| SEN461 | Mata *ade2-101 met2 lys2-801 ura3-52 trp1::hisG leu2∆ his3∆200*  *CEN::URA3:: ∆ssCPY*-MYC* | This study | |
| SEN463 | Mata *ade2-101 met2 lys2-801 ura3-52 trp1::hisG leu2∆ his3∆200 dfm1∆::KanMX*  *CEN::URA3:: ∆ssCPY*-MYC* | This study | |
| SEN464 | Mata *ADE2 met15∆0 LYS2 (LYS+) ura3∆0 TRP1 leu2∆0 his3∆1 ubp14∆::KanMX*  *CEN::URA3:: ∆ssCPY*-MYC* | This study | |
| SEN449 | Mata *ADE2 met15∆0 LYS2 (LYS+) ura3∆0 TRP1 leu2∆0 his3∆1 ubp6∆::KanMX*  *CEN::URA3* | This study | |
| SEN450 | Mata *ADE2 met15∆0 LYS2 (LYS+) ura3∆0 TRP1 leu2∆0 his3∆1 ubp14∆::KanMX*  *CEN::URA3* | This study | |
| SEN451 | Mata *ade2-101 met2 lys2-801 ura3-52 trp1::hisG leu2∆ his3∆200 dfm1∆::KanMX*  *CEN::URA3* | This study | |
| SEN215 | *Mata ADE2::URA3::pTDH3-HMG2-GFPx met15∆0 LYS2(LYS+) ura3∆0 TRP1 leu2∆0 his3∆1 dfm1∆::KanMX*  *CEN::LEU2::pDFM1-DFM1-3HA-L64V* | Nejatfard, et al., 2021 | |
| SEN216 | *Mata ADE2::URA3::pTDH3-HMG2-GFPx met15∆0 LYS2(LYS+) ura3∆0 TRP1 leu2∆0 his3∆1 dfm1∆::KanMX*  *CEN::LEU2::pDFM1-DFM1-3HA-F107S* | Nejatfard, et al., 2021 | |
| SEN217 | *Mata ADE2::URA3::pTDH3-HMG2-GFPx met15∆0 LYS2(LYS+) ura3∆0 TRP1 leu2∆0 his3∆1 dfm1∆::KanMX*  *CEN::LEU2::pDFM1-DFM1-3HA-K67E* | Nejatfard, et al., 2021 | |
| SEN218 | *Mata ADE2::URA3::pTDH3-HMG2-GFPx met15∆0 LYS2(LYS+) ura3∆0 TRP1 leu2∆0 his3∆1 dfm1∆::KanMX*  *CEN::LEU2::pDFM1-DFM1-3HA-Q101R* | Nejatfard, et al., 2021 | |
| SEN219 | *Mata ADE2::URA3::pTDH3-HMG2-GFPx met15∆0 LYS2(LYS+) ura3∆0 TRP1 leu2∆0 his3∆1 dfm1∆::KanMX*  *CEN::LEU2::pDFM1-DFM1-3HA-F58S* | Nejatfard, et al., 2021 | |
| SEN529 | *Mata ADE2::URA3::pTDH3-HMG2-GFPx met15∆0 LYS2(LYS+) ura3∆0 TRP1 leu2∆0 his3∆1 dfm1∆::KanMX*  *CEN::LEU2* | Nejatfard, et al., 2021 | |
| SEN530 | *Mata ADE2::URA3::pTDH3-HMG2-GFPx met15∆0 LYS2(LYS+) ura3∆0 TRP1 leu2∆0 his3∆1 dfm1∆::KanMX*  *CEN::LEU2::pDFM1-DFM1-3HA-AR* | Nejatfard, et al., 2021 | |
| SEN532 | *Mata ADE2::URA3::pTDH3-HMG2-GFPx met15∆0 LYS2(LYS+) ura3∆0 TRP1 leu2∆0 his3∆1 dfm1∆::KanMX*  *CEN::LEU2::pDFM1-DFM1-3HA-AxxxG* | Nejatfard, et al., 2021 | |
| SEN534 | *Mata ADE2::URA3::pTDH3-HMG2-GFPx met15∆0 LYS2(LYS+) ura3∆0 TRP1 leu2∆0 his3∆1 dfm1∆::KanMX*  *CEN::LEU2::pDFM1-DFM1-3HA* | Nejatfard, et al., 2021 | |
| SEN535 | *Mata ADE2::URA3::pTDH3-HMG2-GFPx met15∆0 LYS2(LYS+) ura3∆0 TRP1 leu2∆0 his3∆1 dfm1∆::KanMX*  *CEN::LEU2::pDFM1-DFM1-3HA-5aShp* | Nejatfard, et al., 2021 | |
| SEN506 | Mata *ade2::ADE2::HIS3 met2 lys2-801 ura3-52, trp1::hisG leu2::LEU2::ADE2:: pADH1-Derlin-1-MYC his3∆200 dfm1∆::KanMX* | This study | |
| SEN507 | Mata *ade2::ADE2::HIS3 met2 lys2-801 ura3-52, trp1::hisG leu2::LEU2::ADE2:: pADH1-Derlin-1-MYC his3∆200* | This study | |
| SEN510 | Mata *ade2::ADE2::HIS3 met2 lys2-801 ura3-52, trp1::hisG leu2::LEU2::ADE2:: pADH1-Derlin-2-MYC his3∆200* | This study | |
| SEN512 | Mata *ade2::ADE2::HIS3::pGAL1::Hmg2-GFP met2 lys2-801 ura3-52, trp1::hisG leu2::LEU2::ADE2::pADH1-Derlin-2-MYC his3∆200 dfm1∆::KanMX* | This study | |
| SEN515 | Mata *ade2::ADE2::HIS3::pGAL1::Hmg2-GFP met2 lys2-801 ura3-52, trp1::hisG leu2::LEU2::ADE2 his3∆200 dfm1∆::KanMX* | This study | |
| SEN516 | Mata *ade2::ADE2::HIS3::pGAL1::Hmg2-GFP met2 lys2-801 ura3-52, trp1::hisG leu2::LEU2::ADE2 his3∆200* | This study | |
| SEN470 | Mata *ade2::ADE2::HIS3 met2 lys2-801 ura3-5, trp1::hisG leu2∆ his3∆200*  *CEN::URA3::CFTR-HA* | This study | |
| SEN472 | Mata *ade2::ADE2::HIS3 met2 lys2-801 ura3-52, trp1::hisG leu2::LEU2::ADE2 his3∆200 dfm1∆::KanMX*  *CEN::URA3::CFTR-HA* | This study | |
| SEN474 | Mata *ade2::ADE2::HIS3 met2 lys2-801 ura3-5, trp1::hisG leu2∆ his3∆200*  *CEN::URA3::CFTR-HA-∆F508* | This study | |
| SEN476 | Mata *ade2::ADE2::HIS3 met2 lys2-801 ura3-52, trp1::hisG leu2::LEU2::ADE2 his3∆200 dfm1∆::KanMX*  *CEN::URA3::CFTR-HA-∆F508* | This study | |
| SEN478 | Mata *ade2::ADE2::HIS3 met2 lys2-801 ura3-5, trp1::hisG leu2∆ his3∆200*  *CEN::URA3::A1PiZ* | This study | |
| SEN480 | Mata *ade2::ADE2::HIS3 met2 lys2-801 ura3-52, trp1::hisG leu2::LEU2::ADE2 his3∆200 dfm1∆::KanMX*  *CEN::URA3::A1PiZ* | This study | |
| SEN452 | Mata *ade2::ADE2::HIS3 met2 lys2-801 ura3-52, trp1::hisG leu2::LEU2::ADE2 his3∆200 dfm1∆::KanMX*  *CEN::URA3* | This study | |
| SEN455 | Mata *ade2::ADE2::HIS3 met2 lys2-801 ura3-52, trp1::hisG leu2::LEU2::ADE2 his3∆200*  *CEN::URA3* | This study | |
| SEN365 | ata *ADE2::HIS3::pGAL1::Hmg2-GFP met15∆0 LYS2 (LYS+) ura3∆0 TRP1 leu2∆0 his3∆1 pdr1∆::KanMX* | This study | |
| SEN366 | Mata *ADE2::HIS3 met15∆0 LYS2 (LYS+) ura3∆0 TRP1 leu2∆0 his3∆1 pdr1∆::KanMX* | This study | |
| SEN395 | Mata *ADE2::HIS3::pGAL1::Hmg2-GFP met15∆0 LYS2 (LYS+) ura3∆0 TRP1 leu2∆0 his3∆1 ubp8∆::KanMX* | This study | |
| SEN418 | Mata *ADE2::HIS3 met15∆0 LYS2 (LYS+) ura3∆0 TRP1 leu2∆0 his3∆1 ubp8∆::KanMX* | This study | |
| SEN396 | Mata *ADE2::HIS3::pGAL1::Hmg2-GFP met15∆0 LYS2 (LYS+) ura3∆0 TRP1 leu2∆0 his3∆1 miy2∆::KanMX* | This study | |
| SEN419 | Mata *ADE2::HIS3 met15∆0 LYS2 (LYS+) ura3∆0 TRP1 leu2∆0 his3∆1 miy2∆::KanMX* | This study | |
| SEN397 | Mata *ADE2::HIS3::pGAL1::Hmg2-GFP met15∆0 LYS2 (LYS+) ura3∆0 TRP1 leu2∆0 his3∆1 otu2∆::KanMX* | This study | |
| SEN420 | Mata *ADE2::HIS3 met15∆0 LYS2 (LYS+) ura3∆0 TRP1 leu2∆0 his3∆1 otu2∆::KanMX* | This study | |
| SEN398 | Mata *ADE2::HIS3::pGAL1::Hmg2-GFP met15∆0 LYS2 (LYS+) ura3∆0 TRP1 leu2∆0 his3∆1 ubp2∆::KanMX* | This study | |
| SEN421 | Mata *ADE2::HIS3 met15∆0 LYS2 (LYS+) ura3∆0 TRP1 leu2∆0 his3∆1 ubp2∆::KanMX* | This study | |
| SEN399 | Mata *ADE2::HIS3::pGAL1::Hmg2-GFP met15∆0 LYS2 (LYS+) ura3∆0 TRP1 leu2∆0 his3∆1 ubp5∆::KanMX* | This study | |
| SEN422 | Mata *ADE2::HIS3 met15∆0 LYS2 (LYS+) ura3∆0 TRP1 leu2∆0 his3∆1 ubp5∆::KanMX* | This study | |
| SEN400 | Mata *ADE2::HIS3::pGAL1::Hmg2-GFP met15∆0 LYS2 (LYS+) ura3∆0 TRP1 leu2∆0 his3∆1 miy1∆::KanMX* | This study | |
| SEN423 | Mata *ADE2::HIS3 met15∆0 LYS2 (LYS+) ura3∆0 TRP1 leu2∆0 his3∆1 miy1∆::KanMX* | This study | |
| SEN402 | Mata *ADE2::HIS3::pGAL1::Hmg2-GFP met15∆0 LYS2 (LYS+) ura3∆0 TRP1 leu2∆0 his3∆1 ubp1∆::KanMX* | This study | |
| SEN425 | Mata *ADE2::HIS3 met15∆0 LYS2 (LYS+) ura3∆0 TRP1 leu2∆0 his3∆1 ubp1∆::KanMX* | This study | |
| SEN403 | Mata *ADE2::HIS3::pGAL1::Hmg2-GFP met15∆0 LYS2 (LYS+) ura3∆0 TRP1 leu2∆0 his3∆1 ubp11∆::KanMX* | This study | |
| SEN426 | Mata *ADE2::HIS3 met15∆0 LYS2 (LYS+) ura3∆0 TRP1 leu2∆0 his3∆1 ubp11∆::KanMX* | This study | |
| SEN405 | Mata *ADE2::HIS3::pGAL1::Hmg2-GFP met15∆0 LYS2 (LYS+) ura3∆0 TRP1 leu2∆0 his3∆1 ubp7∆::KanMX* | This study | |
| SEN428 | Mata *ADE2::HIS3 met15∆0 LYS2 (LYS+) ura3∆0 TRP1 leu2∆0 his3∆1 ubp7∆::KanMX* | This study | |
| SEN406 | Mata *ADE2::HIS3::pGAL1::Hmg2-GFP met15∆0 LYS2 (LYS+) ura3∆0 TRP1 leu2∆0 his3∆1 ubp3∆::KanMX* | This study | |
| SEN429 | Mata *ADE2::HIS3 met15∆0 LYS2 (LYS+) ura3∆0 TRP1 leu2∆0 his3∆1 ubp3∆::KanMX* | This study | |
| SEN453 | Mata *ade2::ADE2::HIS3 met2 lys2-801 ura3-52, trp1::hisG leu2∆ his3∆200*  *CEN::URA3::pCUP1-HBT-Ubiquitin* | This study | |
| SEN454 | Mata *ade2::ADE2::HIS3 met2 lys2-801 ura3-52, trp1::hisG leu2∆ his3∆200*  *CEN::URA3* | This study | |
| SEN457 | Mata *ade2::ADE2::HIS3 met2 lys2-801 ura3-52, trp1::hisG leu2∆ his3∆200*  *CEN::URA3::pCUP1-HBT-Ubiquitin* | This study | |
| SEN455 | Mata *ade2::ADE2::HIS3 met2 lys2-801 ura3-52, trp1::hisG leu2∆ his3∆200*  *CEN::URA3* | This study | |
| SEN481 | Mata *ade2::ADE2::HIS3::pGAL1::Hmg2-GFP met2 lys2-801 ura3-52, trp1::hisG leu2∆ his3∆200 dfm1∆::KanMX*  *CEN::URA3::pCUP1-HBT-Ubiquitin* | This study | |
| SEN456 | Mata *ade2::ADE2::HIS3::pGAL1::Hmg2-GFP met2 lys2-801 ura3-52, trp1::hisG leu2∆ his3∆200 dfm1∆::KanMX*  *CEN::URA3* | This study | |
| SEN482 | Mata *ade2::ADE2::HIS3 met2 lys2-801 ura3-52, trp1::hisG leu2∆ his3∆200 dfm1∆::KanMX*  *CEN::URA3::pCUP1-HBT-Ubiquitin* | This study | |
| SEN452 | Mata *ade2::ADE2::HIS3 met2 lys2-801 ura3-52, trp1::hisG leu2∆ his3∆200 dfm1∆::KanMX*  *CEN::URA3* | This study | |
| SEN122 | *ade2::ADE2::HIS3 met2 lys2-801 ura3-52::URA3::4xUPRE::GFP TRP1 leu2∆ his3∆200 der1∆::CloNat* | This study | |
| SEN123 | *ade2::ADE2::HIS3::pGAL::Hmg2-6MYC met2 lys2-801 ura3-52::URA3::4xUPRE::GFP TRP1 leu2∆his3∆200 der1∆::CloNat* | This study | |
| RHY11923 | Mata *ade2-101 met2 lys2-801 ura3-5, trp1::hisG::pGAL::Hmg2-GFP leu2∆ his3∆200 pdr5∆::HIS3* | This study | |
| RHY11924 | Mata *ade2-101 met2 lys2-801 ura3-5, trp1::hisG::pGAL::Hmg2-GFP leu2∆ his3∆200 pdr5∆::HIS3*  *hrd1∆::KanMX* | This study | |
| RHY11925 | Mata *ade2-101 met2 lys2-801 ura3-5, trp1::hisG::pGAL::Hmg2-GFP leu2∆ his3∆200 pdr5∆::HIS3*  *dfm1∆::CloNAT* | This study | |
| RHY11916 | Mata *ade2-101 met2 lys2-801 ura3-5, trp1::hisG::TRP1 leu2∆ his3∆200 pdr5∆::HIS3* | This study | |
| RHY11917 | Mata *ade2-101 met2 lys2-801 ura3-5, trp1::hisG::TRP1 leu2∆ his3∆200 pdr5∆::HIS3*  *hrd1∆::KanMX* | This study | |
| RHY11918 | Mata *ade2-101 met2 lys2-801 ura3-5, trp1::hisG::TRP1 leu2∆ his3∆200 pdr5∆::HIS3*  *dfm1∆::CloNAT* | This study | |
| SEN409 | Mata *ade2 met2 lys2-801 ura3-52 trp1::hisG leu2∆ his3∆200 dfm1∆::KanMX*  *CEN::URA3::CPY*-GFP* | Nejatfard, et al., 2021 | |
| SEN410 | Mata *ade2 met2 lys2-801 ura3-52 trp1::hisG leu2∆ his3∆200 dfm1∆::KanMX*  *CEN::URA3::CPY*-GFP*  *CEN::LEU2::pDFM1-DFM1-3HA* | Nejatfard, et al., 2021 | |
| SEN206 | *Mata ADE2 met15∆0 LYS2(LYS+) ura3∆0 TRP1 leu2∆0 his3∆1 hrd1∆::KanMX*  *CEN::HIS3::PDR5*-HA*  *CEN::LEU2::pDFM1-DFM1-3HA* | Nejatfard, et al., 2021 | |
| SEN264 | *Mata ADE2 met15∆0 LYS2(LYS+) ura3∆0 TRP1 leu2∆0 his3∆1 hrd1∆::KanMX*  *CEN::HIS3::PDR5*-HA*  *CEN::LEU2* | Nejatfard, et al., 2021 | |
| SEN554 | *ade2-101 met2 lys2-801 ura3-52::URA3::HMG2-GFP trp1::hisG leu2∆ his3∆200 pdr5∆::KanMX* | This study | |
| SEN555 | *ade2-101 met2 lys2-801 ura3-52::URA3::HMG2-GFP trp1::hisG::TRP1 leu2∆ his3∆200 hrd1∆::KanMX* | This study | |
| SEN556 | *ade2-101 met2 lys2-801 ura3-52::URA3::HMG2-GFP trp1::hisG::TRP1::pHrd1::Hrd1-5MYC leu2∆ his3∆200 hrd1∆::KanMX* | This study | |
| RHY10584 | Mata *ADE2::ADE2::URA3::TDH3p-HMG2-GFP-K6R met15∆0 LYS2 (LYS+) ura3∆0 TRP1 leu2∆0 his3∆1 dfm1∆::KanMX* | This study | |
| RHY12239 | \| *ade2-101 met2 lys2-801 ura3-52::URA3::HMG2-GFP trp1::hisG::TRP1 leu2∆ his3∆200 dfm1∆::KanMX* \| \| --- \| \| *CEN::LEU2* \| | This study | |
| RHY12240 | \| *ade2-101 met2 lys2-801 ura3-52 trp1::hisG::TRP1::TDH3-SEC61-GFP leu2∆ his3∆200 dfm1∆::KanMX* \| \| --- \| \| *CEN::LEU2::pDFM1-DFM1-3HA* \| | This study | |
| RHY12241 | \| *ade2-101 met2 lys2-801 ura3-52 trp1::hisG::TRP1::TDH3-SEC61-GFP leu2∆ his3∆200 dfm1∆::KanMX* \| \| --- \| \| *CEN::LEU2::pDFM1-5ASHP-3HA* \| | This study | |

**References**

1. Neal S, Jaeger PA, Duttke SH, Benner CK, Glass C, Ideker T, et al. The Dfm1 Derlin Is Required for ERAD Retrotranslocation of Integral Membrane Proteins. Mol Cell. 2018;69(2).
2. Neal S, Syau D, Nejatfard A, Nadeau S, Hampton RY. HRD Complex Self-Remodeling Enables a Novel Route of Membrane Protein Retrotranslocation. iScience. 2020;23(9).
3. Nejatfard A, Wauer N, Bhaduri S, Conn A, Gourkanti S, Singh N, et al. Derlin rhomboid pseudoproteases employ substrate engagement and lipid distortion to enable the retrotranslocation of ERAD membrane substrates. Cell Rep. 2021;37(3):109840.
