# Supplementary material for: Yeast derlin Dfm1 employs a chaperone-like function to resolve misfolded membrane protein stress
Source: PLoS Biol. 2023 Jan 23;21(1):e3001950. doi: 10.1371/journal.pbio.3001950 (PMC9894555; doi:10.1371/journal.pbio.3001950)
Supplement: S1 Raw images — (PDF) [file pbio.3001950.s010.pdf]

Figure 3A and 3 B Top Panel

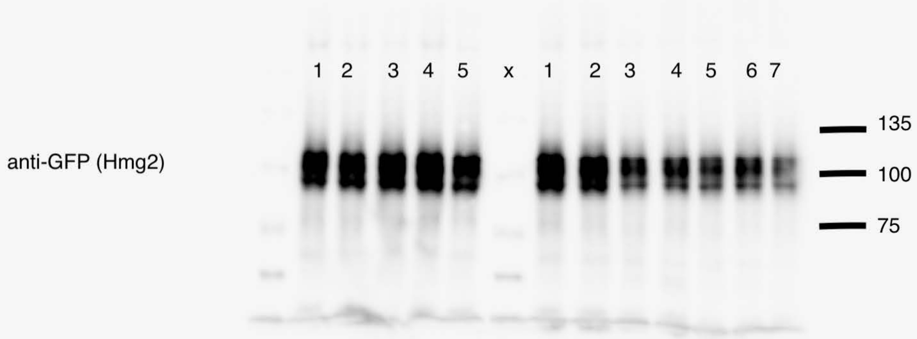

Image acquisition: Western blot was acquired using ChemiDoc on chemiluminescence setting. Image was exported in high resolution (300 dpi) in TIF file format.

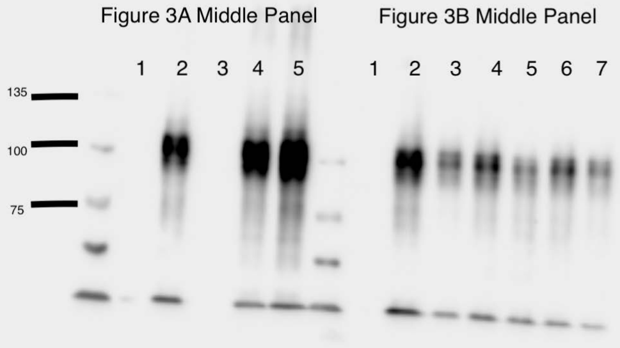

Image acquisition: Western blot was acquired using ChemiDoc on chemiluminescence setting. Image was exported in high resolution (300 dpi) in TIF file format.

anti-GFP (Hmg2)

Figure 3A and 3B Bottom Panel

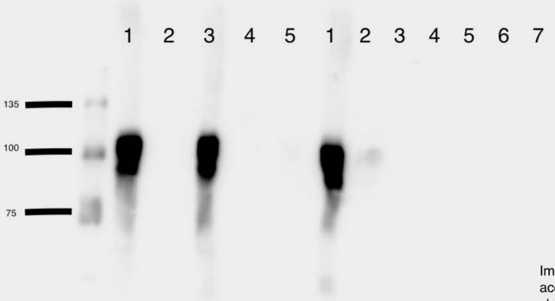

anti-GFP (Hmg2)

Image acquisition: Western blot was acquired using ChemiDoc on chemiluminescence setting. Image was exported in high resolution (300 dpi) in TIF file format.

Fig. 3C

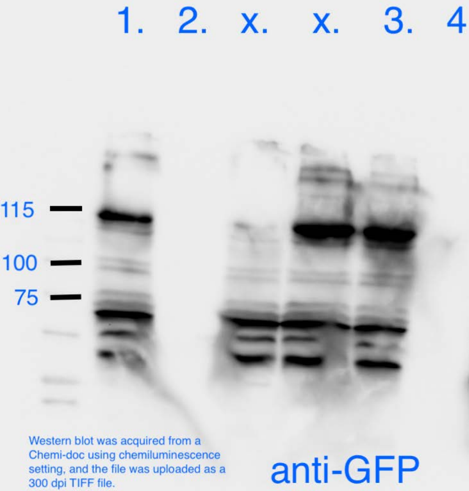

Western blot was acquired from a Chemi-doc using chemiluminescence setting, and the file was uploaded as a 300 dpi TIFF file.

Fig. 3C

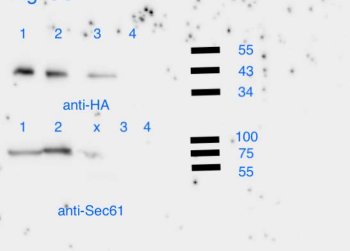

Image acquisition: Western blot was acquired from a Chemi-Doc, using a chemiluminescence setting, and the file was uploaded as a TIFF file format.

Figure 4A

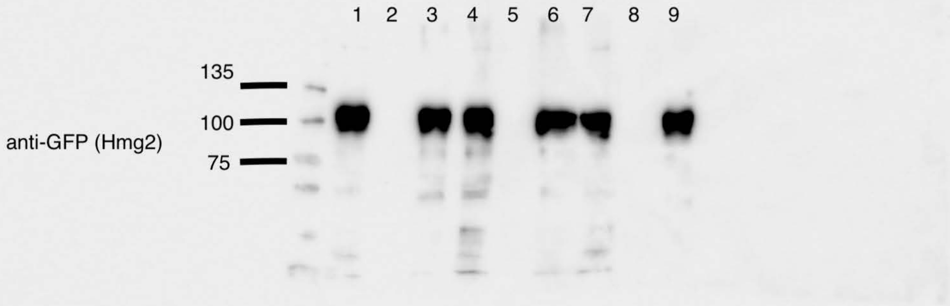

Image acquisition: Western blot was acquired using ChemiDoc on chemiluminescence setting. Image was exported in high resolution (300 dpi) in TIF file format.

Fig. 4B

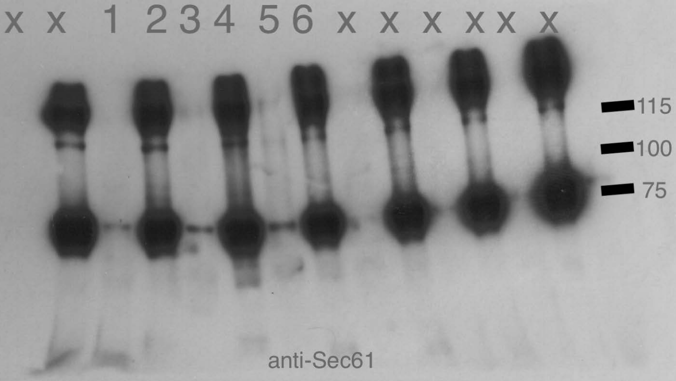

Image acquisition: Western blot was acquired from a Chemi-doc, using a chemiluminescence setting, and the file was updated as a 300 dpi TIFF file.

Figure 4C

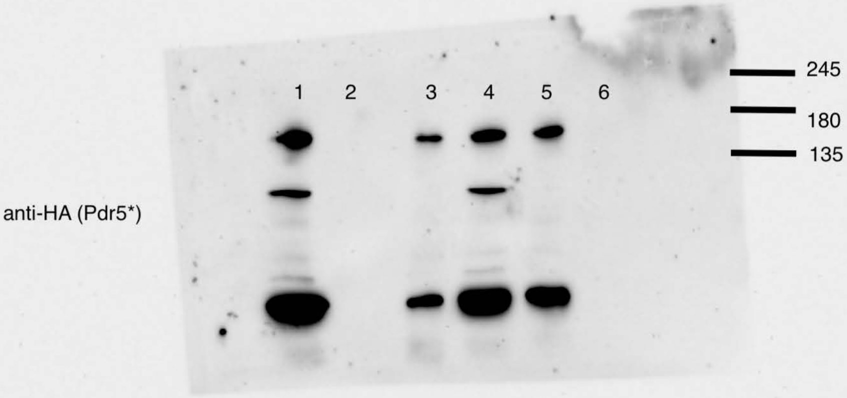

Image acquisition: Western blot was acquired using ChemiDoc on chemiluminescence setting. Image was exported in high resolution (300 dpi) in TIF file format.

Figure 4E

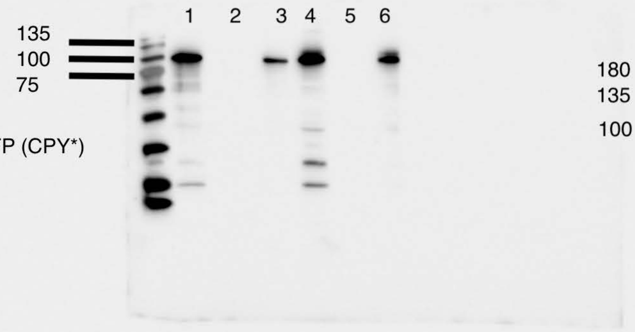

anti-GFP (CPY*)

Figure 4D

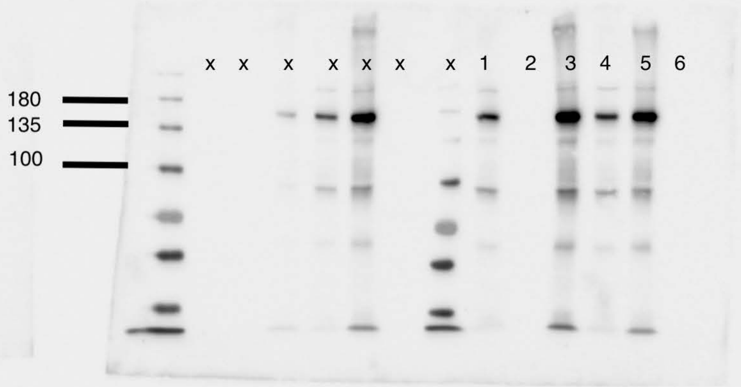

anti-GFP (Ste6*)

Image acquisition: Western blot was acquired using ChemiDoc on chemiluminescence setting. Image was exported in high resolution (300 dpi) in TIF file format.

Figure 4F

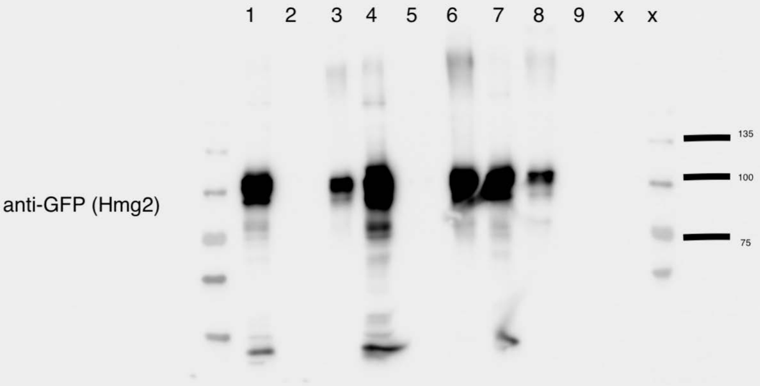

Image acquisition: Western blot was acquired using ChemiDoc on chemiluminescence setting. Image was exported in high resolution (300 dpi) in TIF file format.

Figure 6G Top Panel

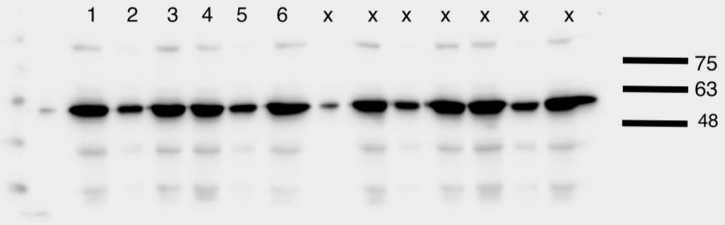

anti-GFP (Pre6)

Image acquisition: Western blot was acquired using ChemiDoc on chemiluminescence setting. Image was exported in high resolution (300 dpi) in TIF file format.

Fig. 7B (left panel)

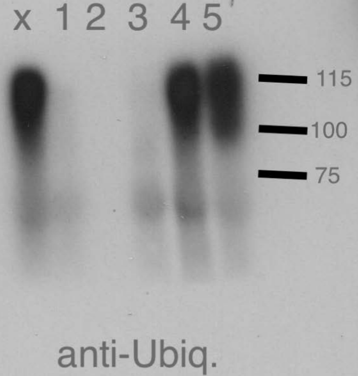

Western blot was acquired from an automatic film processor.
The film was scanned in high resolution (600 dpi) in TIFF format.

Fig. 7B (left panel)

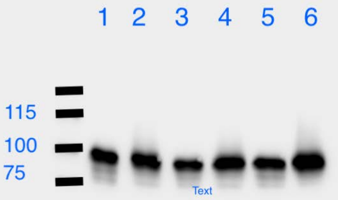

anti-GFP

Western blot was acquired from an automatic film processor.
The film was scanned in high resolution (600 dpi) in TIFF format.

Figure 7B (right panel

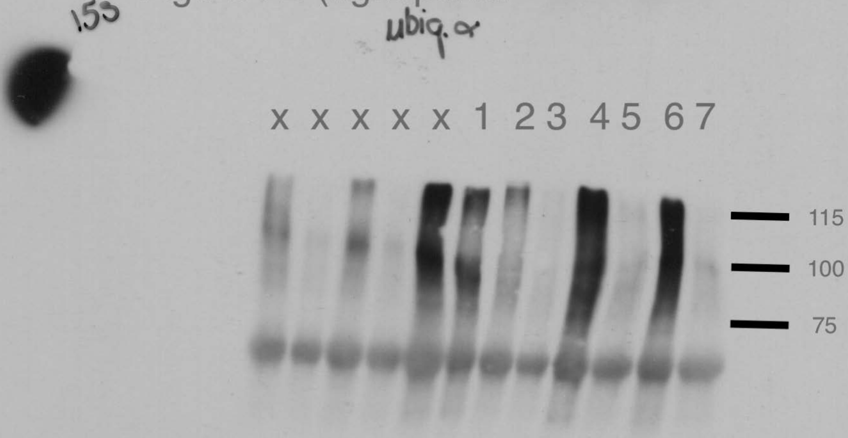

anti-ubiq.

Western blot was acquired from an automatic film processor.
The film was scanned in high resolution (600 dpi) in TIFF format.

Fig. 7B (right panel)

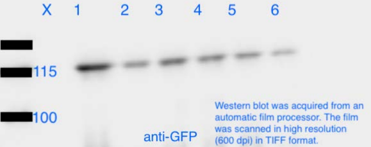

Figure 7E top panel

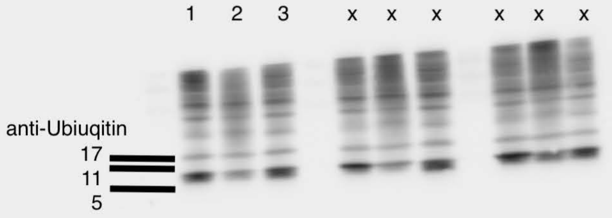

Image acquisition: Western blot was acquired using ChemiDoc on chemiluminescence setting.

Image was exported in high resolution (300 dpi) in TIF file format.

Figure 7E Bottom Panel

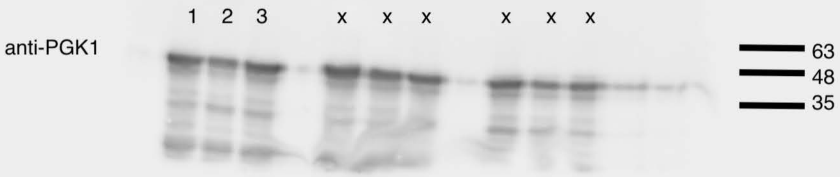

Image acquisition: Western blot was acquired using ChemiDoc on chemiluminescence setting. Image was exported in high resolution (300 dpi) in TIF file format.

Figure 6G Bottom Panel

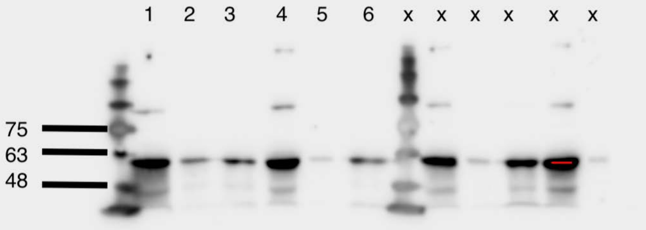

anti-GFP (Pre6)

Image acquisition: Western blot was acquired using ChemiDoc on chemiluminescence setting. Image was exported in high resolution (300 dpi) in TIF file format.

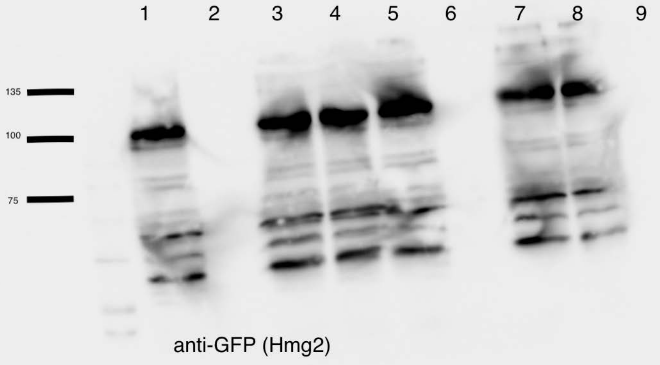

Image acquisition: Western blot was acquired using ChemiDoc on chemiluminescence setting. Image was exported in high resolution (300 dpi) in TIF file format.

Figure 7I

Figure S1C (Left panel)

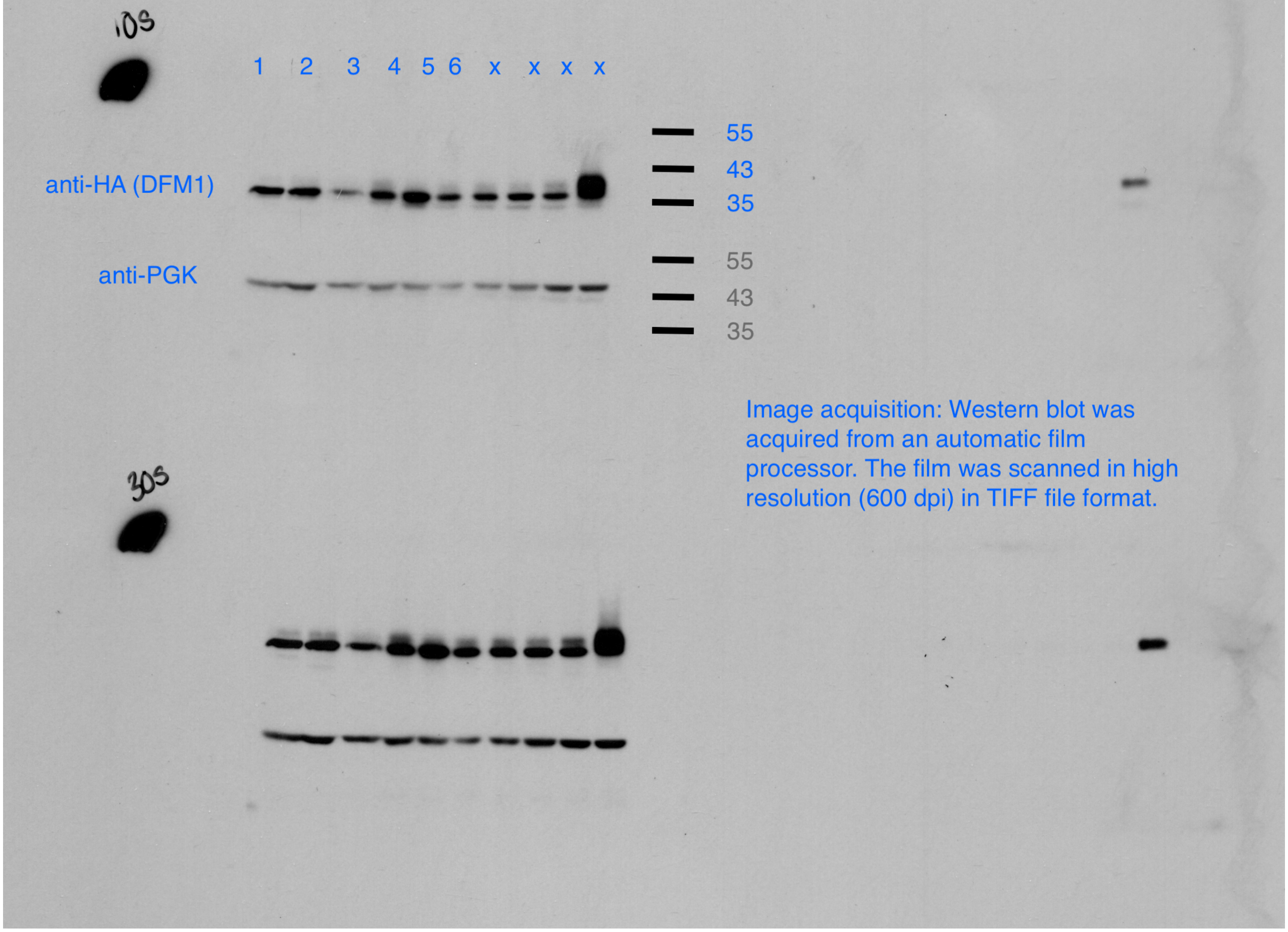

Image acquisition: Western blot was acquired from an automatic film processor. The film was scanned in high resolution (600 dpi) in TIFF file format.

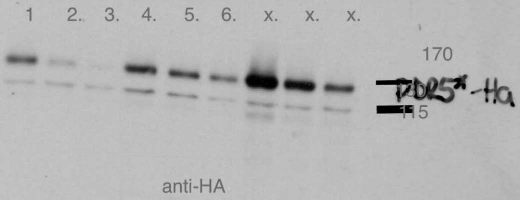

Fig. S1A Top panel

Western blot was acquired from an automatic film processor. The film was scanned in high resolution (600 dpi) TIFF file format.

Fig. S1A Bottom Panel

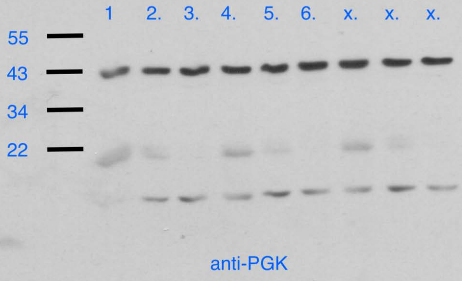

Western blot was acquired from an automatic film processor. The film was scanned in high resolution (600 dpi) TIFF file format.

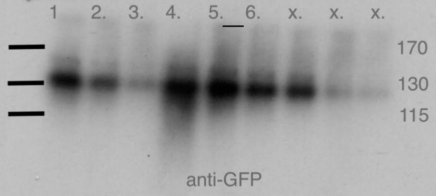

Fig. S1B Top panel

Western blot was acquired from an automatic film processor. The film was scanned in high resolution (600 dpi) TIFF file format.

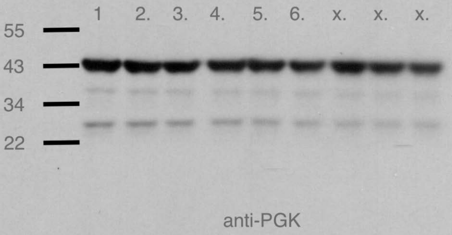

Fig. S1B Bottom Panel

Western blot was acquired from an automatic film processor. The film was scanned in high resolution (600 dpi) TIFF file format.

Figure S1C (Left panel)

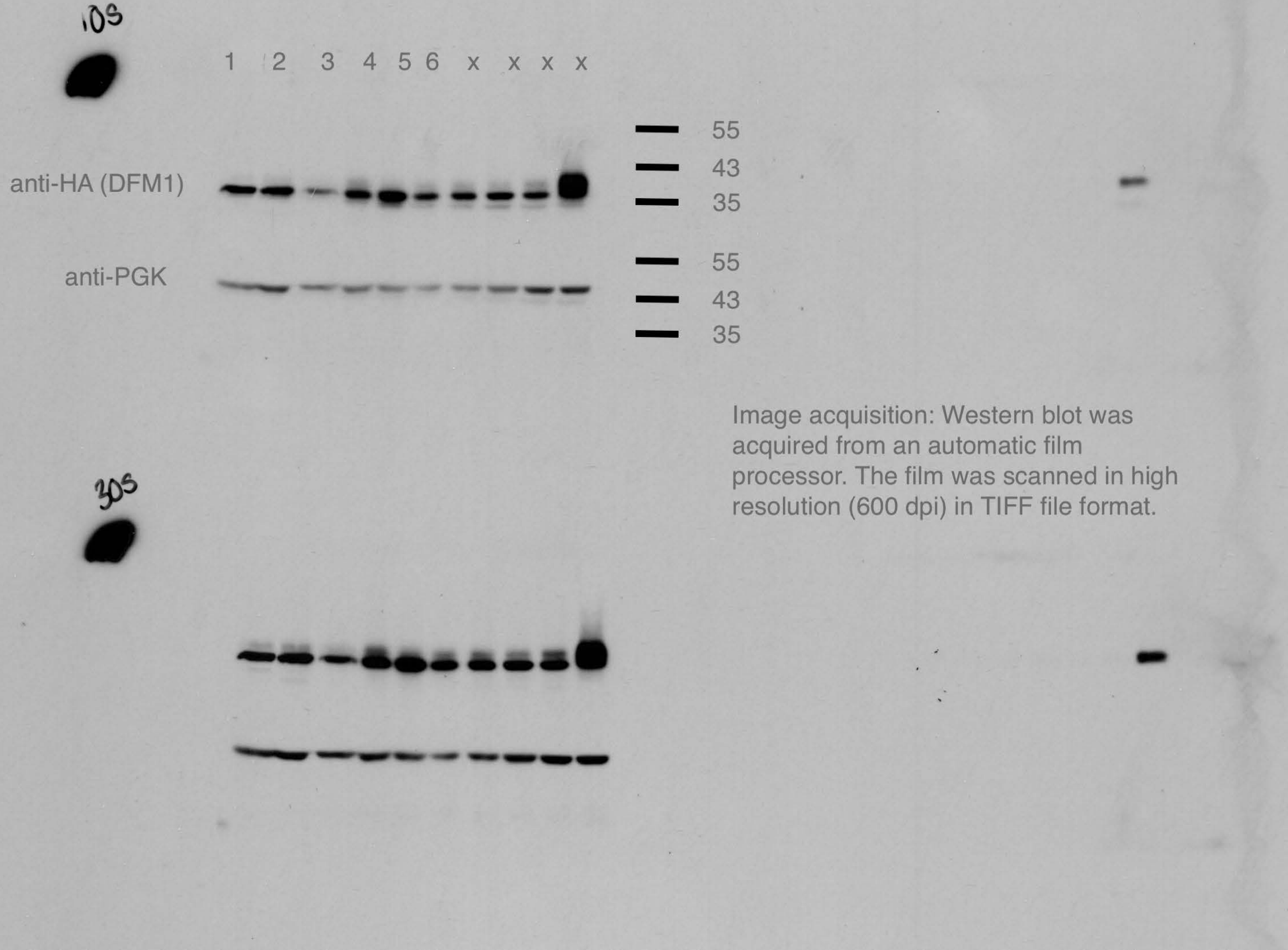

Image acquisition: Western blot was acquired from an automatic film processor. The film was scanned in high resolution (600 dpi) in TIFF file format.

Figure S1C Top Panel (Right)

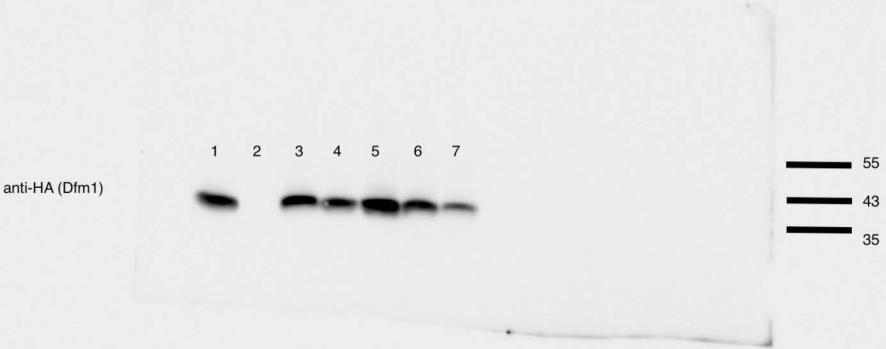

Image acquisition: Western blot was acquired using ChemiDoc on chemiluminescence setting. Image was exported in high resolution (300 dpi) in TIF file format.

Figure S1C Bottom Panel (Right)

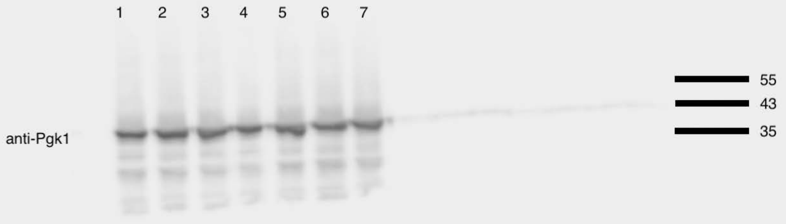

Image acquisition: Western blot was acquired using ChemiDoc on chemiluminescence setting. Image was exported in high resolution (300 dpi) in TIF format.

Figure S2B

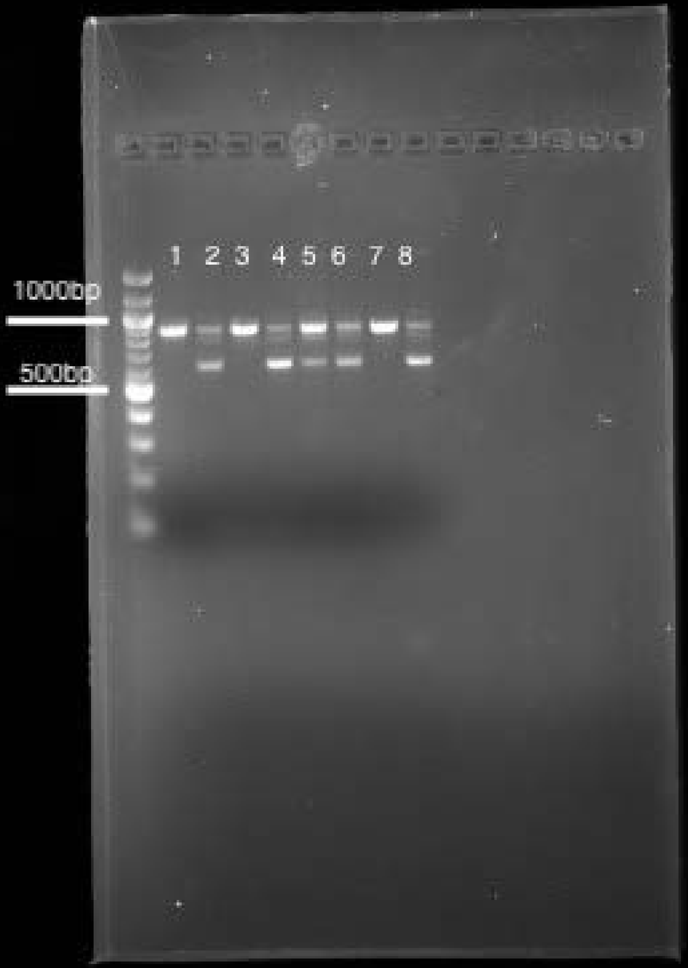

Figure S2A

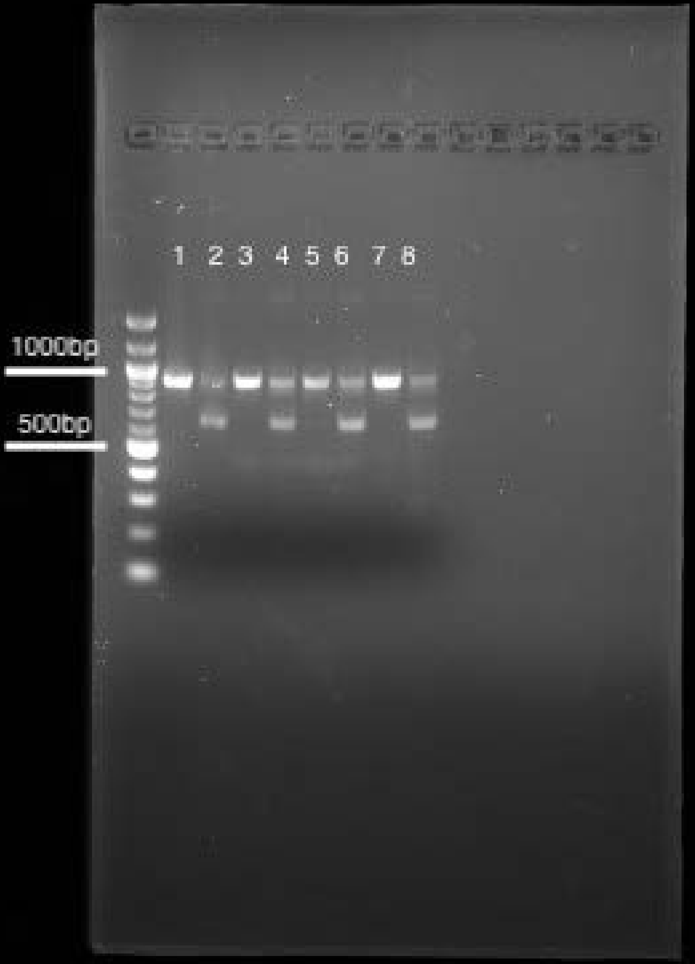

Image acquisition: DNA gel was acquired using ChemiDoc on SYBR Safe setting. Image was exported in high resolution (300 dpi) in TIF format.

Figure S5A Top Panel

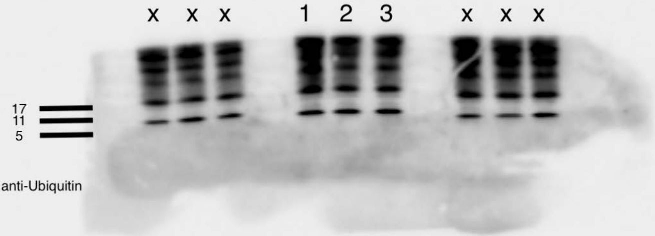

Image acquisition: Western blot was acquired using ChemiDoc on chemiluminescence setting. Image was exported at high resolution (300 dpi) in TIF format.

Figure S5A Bottom Panel

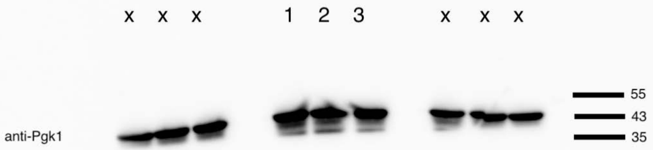

Image acquisition: Western blot was acquired using ChemiDoc on chemiluminescence setting. Image was exported at high resolution (300 dpi) in TIF format.
