# Supplementary material for: Yeast derlin Dfm1 employs a chaperone-like function to resolve misfolded membrane protein stress
Source: PLoS Biol. 2023 Jan 23;21(1):e3001950. doi: 10.1371/journal.pbio.3001950 (PMC9894555; doi:10.1371/journal.pbio.3001950)
Supplement: Attachment — Submitted filename: PLOS Biology Reviews.pdf [file pbio.3001950.s012.pdf]

Rev. 1:
In this manuscript submitted to PLOS Biology (PBIOLOGY-D-22-01696R1), Kandel et al. examine the contribution of the S. cerevisiae Rhomboid pseudoprotease, Dfm1 in the solubilization of misfolded integral membrane proteins. The role of Dfm1 in preparing integral membrane substrates for retrotranslocation via substrate engagement, membrane thinning, and Cdc48 recruitment has been established by a series of well-executed studies. Using a substrate toxicity assay and a plethora of constructs/tools to study Dfm1 function, growth assays, and Western Blotting, this report aims to establish a hitherto unappreciated Dfm1 chaperone-like activity. Moreover, while it has long been known that the expression of misfolded integral membrane proteins can cause toxicity in certain yeast genetic backgrounds (knockout strains), the mechanism has remained elusive. Therefore, in this manuscript, using genetic tools, the authors try to dissect the cellular event that is eliciting the toxicity which is specific to integral membrane ERAD substrates but not the lumenal substrate (CPY*). Overall, the data are presented in a clear and logical fashion and the quality of the data is sufficient to make the arguments convincing. The paper presents new and surprising data on all fronts and will be an excellent addition to the field. It was very pleasing to read, as it methodically answered each new question opened by the previous paragraph. The study also raised new and interesting questions to explore. There are just a few minor concerns with figures, references, and framing that should be fixed prior to acceptance for publication.

Text Concerns
1. Line 52, more primary references should be added, Bays Nat Cell Bio 2001: Meyer EMBO J 2000: Rabinovich Mol Cell Biol 2002: Ye and Rapoport Nature 2001
Thank you for the suggestion, additional references have been added.

2. Line 25 vs Line 490 clear up the number of integral membrane proteins (should be closer to ¼ as initially stated)
This has been changed to say one quarter in both instances.

3. There should be a reference included for the end of line 73.
Several citations have been added to this.

4. There should be a reference included for the end of 87.
We have added several references (now line 88) and reworded this sentence: “This study is the first to demonstrate chaperone-like activity for any rhomboid protein. Many rhomboid proteins use similar functions as Dfm1 to promote retrotranslocation and the rhomboid protease RHBDL4 has recently been characterized as acting on aggregation-prone substrates.”

5. Line 134, sentence ending ER E3 ligases. A reference should be added or data should be added to the supplement for proof of the statment. Often ERAD substrates can require the action of two or more E3 ligases, so to say that the substrates are not ubiquitinated in single deletions is likely misleading. Perhaps it could be reworded to say reduced ubiquitination…
Thank you for pointing this out. We have added a figure panel (Figure 7B) where we immunoprecipitated Hmg2-GFP or Ste6*-GFP and probed for their ubiquitination status in the

corresponding strains. Indeed, even with single knockouts of the E3 ligases, ubiquitination signal is negligible.

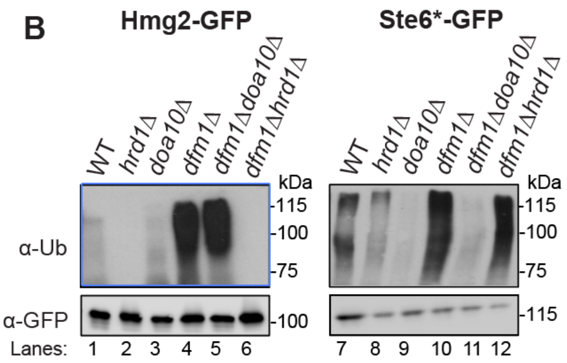

6. Line 299, I do not recall seeing any discussion on what the source of the variable transcriptional changes could be.
The following line has now been added to line 366, "This variability is likely representative of biological variability in these strains rather than experimental variability as it was only observed between the biological replicates and not the technical replicates of the other strains tested."

7. Line 328, reference should be added for Burns FEBS letter 2021, for role of Rpn4.
This citation has been added to the sentence starting on line 409, "This is in line with previous research demonstrating Rpn4 is activated in response to misfolded membrane protein accumulation and that misfolded membrane protein expression can result in proteasome impairment, even in WT cells."

Figure/Experimental concerns

1. Line 137-143. The use of the cdc48-2 allele is clever, but if cdc48 was truly inactivated, then the strain should not divide making the spot test assay impossible. I saw that the permissive temperature of 30 degrees was used, but this is in opposition to most of the papers which use this cdc48 alleles at 37 degrees or higher (Simoes eLife 2018;Gallagher J Cel Sci 2014:Hsieh PLOS One 2011, to name a few) . While it has been shown for Hmg2 that 30 degrees is sufficient to induce an ERAD defect by flow cytometry (Hampton lab) this has not been shown for Pdr5* and Ste6p*. Some language should be added to hedge bets about the impact of the 30 degrees on the substrates. Better yet, if you had some experimental data to show that 30 degrees has an impact for the degradation or retrotranslocation of these substrates, that would be great to include.
   Thank you for suggesting this. We have performed a cycloheximide chase experiment for Pdr5* and Ste6* where each strain was grown at 30ºC and have included this new data as Supplemental Figure 1 A and B (shown below). We have also shown this previously in Neal et al., JBC, 2017 for Pdr5* where retrotranslocation is compromised. In the results section, line 142-147 now reads," To rule out this possibility, we utilized a temperature sensitive Cdc48 allele strain, *cdc48-2,* which, like *dfm1Δ* cells, results in accumulation of ubiquitinated ERAD membrane substrates. While we used *cdc48-2* cells at the permissive temperature of 30°C, ERAD is still compromised for Hmg2, as previously reported, and

we validated using a cycloheximide chase that Pdr5* and Ste6* degradation is also impaired at 30°C in *cdc48-2* cells (Neal, Bennett, and Hampton 2017) (Fig. S1A&B)."

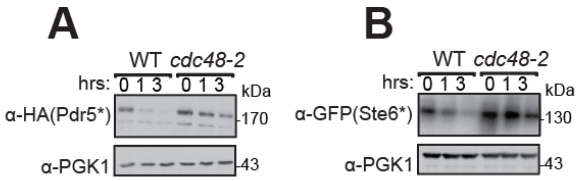

2. It is not unexpected that CFTR and F508del have similar phenotypes as this has been observed by others but not formally published, but in a dissertation somewhere.
Although we could not find the dissertation, we have amended this section in which the beginning of the sentence starting on line 169 that used to read, "While we had originally hypothesized that only CFTRΔF508 would cause a growth defect when expressed in *dfm1Δ* cells," is now changed to, "It was not wholly surprising that WT CFTR also elicited growth stress in *dfm1Δ* cells. Previous studies have shown that while virtually all CFTRΔF508 is targeted to ERAD, about 80% of WT CFTR is degraded via ERAD in yeast and mammals (Cheng et al. 1990; Gnann, Riordan, and Wolf 2004; Zhang et al. 2001)."

3. In Figure 6G and the text starting line 196. You discuss all 3 derlins, but then only show data that two rescued. Was derlin 3 not tested, or did it not rescue? If it was not tested, please state.
We have only tested Derlin-1 and Derlin-2 expression. Derlin-3 has overall not been studied as extensively, and our lab has only cloned Derlin-1 and Derlin-2 into yeast expression vectors. To clarify this, we added this sentence on line 200, "We opted to only test these since Derlin-1 and Derlin-2 have been much more thoroughly researched than Derlin-3."

4. For the detergent solubility assay, it is difficult to know, the data are so clean it is hard to believe! All or nothing. However, a thought occurred as to once the substrate is released from the cell, is Dfm1 still associated with the substrate in DDM, e.g. would they co-IP in this detergent? If that data is available, please include or discuss findings as data not shown. If so, that would explain why Dfm1 can still impart its effect post-lysis. If Dfm1 falls off during the lysis procedure, one must wonder, how did Dfm1 permanently alter the solubility of the substrate, such at it stays in the supe post-lysis?
Thank you for suggesting this forward-thinking experiment! We have done this co-IP experiment and demonstrated that both Hmg2 and Dfm1 are still bound to each other after addition of DDM. This experiment (shown below) has been added as Figure 3C. This sentence was also added on line 267, " This all-or-nothing effect that Dfm1's presence has on aggregation led us to determine whether Dfm1 binds to Hmg2 even after solubilization in with DDM. Indeed, using co-immunoprecipitation, we found that Dfm1 physically interacts with solubilized Hmg2 (Fig. 3C)."

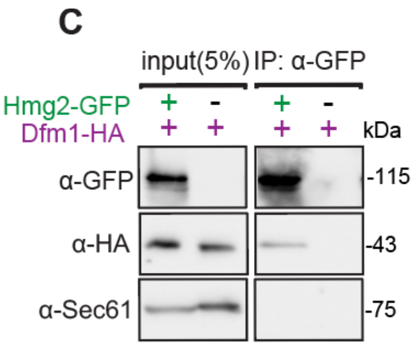

The following was also added to the methods section:

Co-Immunoprecipitation

Yeasts were grown to mid log phase in minimal media, and 15 OD equivalents were pelleted, washed in water, and resuspended in 240 µl lysis buffer (0.24 M sorbitol, 1 mM EDTA, 20 mM KH2PO4/K2HPO4, pH 7.5) with PIs (2 mM phenylmethylsulfonyl fluoride and 142 mM tosylphenylalanyl chloromethyl ketone). Acid-washed glass beads were added up to the meniscus. Cells were lysed on a multivortexer at 4 ºC for six to eight 1-min intervals with 1 min on ice in between each lysis step. The lysates were transferred to a new tube, and lysates cleared with 5-s pulses of centrifugation. Microsomes were pelleted from cleared lysates by centrifugation at 14,000g for 5 min. Microsome pellets were washed once in XL buffer (1.2 M sorbitol, 5 mM EDTA, 0.1 M KH2PO4/K2HPO4, pH 7.5) and resuspended in XL buffer.

Samples were then solubilized by the addition of detergent solution at 10x the desired final concentration in XL buffer (final concentration or 1% DDM). Preparations with detergent were incubated at 4 °C for 1 h with rocking and then repeatedly pipetted up and down. Finally, samples were cleared by centrifugation in a benchtop microcentrifuge for 15 min at 16,000g. The supernatants were then separated by ultracentrifugation at 89,000 RPM for 15 min, and the supernatant from this step was used for the co-immunoprecipitation assay.

5. The flow cytometry data for UPR activation presented in figure S3 is a little confusing to me. In the text you try to make the point that CPY* exacerbates UPR activation in Dfm1 delta cells, but that Hmg2 does not in Der1delta cells. Then I look at the AU between the left column and right column in Fig. S3 and the absolute values can be quite different between the pdr5 strain and the dfm1 strain. It looks like dfm1 knockout is having some impact on baseline ability to activate the UPR because For Ste6p* for example, the pdr5 AU in gal+TM is 3K, and in the dfm1 the gal + TM condition is 30K. Perhaps fold change induction would a better way to display the data. Or some more text to describe why these differences are present and what they mean.

We have removed supplemental Figure 3 and removed a discussion of the figure in the result section that point to CPY* exacerbating stress. This was based on an incomplete analysis that was in an earlier draft of the paper and this error was not noticed before submitting. Our apologies and thank you for catching this issue.

6. Figure 7A, the data for the dfm1delta over expressing Hmg2 and Ste6p* shows growth when in every other figure it shows no growth. This makes the data for this figure difficult to interpret. The figure should be replaced with data that more accurately matches the phenotype present in other growth assays.

We do agree the growth phenotype isn't as strong in this particular case. It seems that there was an issue with this particular growth assay where the strains in one of the replicates had started to suppress the retrotranslocation defect (a phenomenon we have thoroughly investigated in Neal et al., iScience, 2020), or perhaps there was an issue with the galactose plates that did not result in strong expression of substrates. Regardless, we have replaced these images with one another experimental replicate which displayed a much more prominent effect. In addition, we performed another replicate of the experiment to ensure that the mild phenotype was not a persistent issue. The updated panel for Figure 7A is shown below.

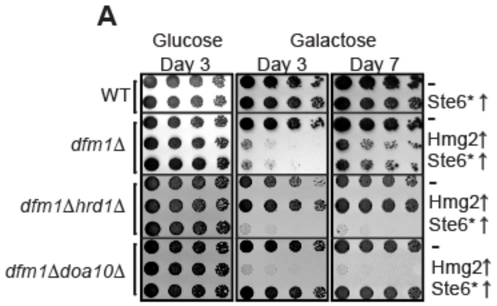

Rev. 2:

This is an interesting manuscript, which describes a comprehensive study that aims to define a new role of Dfm1 in solubilizing aggregates formed by membrane proteins in ER.

This is a follow-up study to one that was published by the same group last year in Cell Reports that identified specific residues in Dfm1 that crucial for the recognition of ubiquitinated ERAD membrane substrates and their retrotranslocation to ER.

In this manuscript, the authors investigate the additional function of Dfm1, which leads to the solubilization of ubiquitinated membrane protein aggregates. This function is Cdc48 independent.

The paper is very well written, however, some of the parts are confusing and the rationale of the experiments as well as their outcomes are not very clear in the context of the whole story. It is especially true for the transcriptomic analysis that was done using different mutant strains than in the rest of the manuscript. In addition, some figures are not cited correctly or missing.

Moreover, the main conclusions of this study are based on growth defect phenotypes shown using dilution spot assays. This is a valid approach in the field, however, since ~80-90% of the main figures are dilution spot assays without any replicates, additional experiments should be provided, such as growth in medium or others.

Thank you for your suggestion. We have added the following sentence to the manuscript on line 858 to clarify that these experiments were done with biological replicates and technical duplicates, “All experiments were done in biological triplicates with two technical replicates.” Additionally, we added this line to the end of every figure legend displaying growth assays, “Data information: All dilution growth assays were performed in 3 biological and 2 technical replicates (N=3).”

The main conclusion is that Dfm1 prevents the formation of aggregates formed by membrane proteins, substrates of ERAD, is solid and well proven by the phenotypic and biochemical study, including solubility test and UPR activation analysis.

However, the second statement that dfm1 deletion "resulting in compromised proteasome function and a depletion of monomeric ubiquitin" is based mainly on phenotypic studies and Figs 7D-E only, which shows not a very significant difference in mono-Ubiquitin.

We appreciate your feedback. While a ~50% decrease in monomeric ubiquitin may not seem biologically significant, there is precedent for this sort of reduction causing massive issues to cellular health. For example, in the paper we cited on mice with a Usp14 mutation that causes a reduction to monomeric ubiquitin and results in ataxia, monomeric ubiquitin was only reduced by about ~25% in all tissues tested (Anderson et al. 2005). Further, this same paper showed a similar reduction in Ubp6 null yeast cells, which are well established as having a reduced pool of ubiquitin and experiencing ubiquitin stress (Anderson et al. 2005; Hanna et al. 2007). Additionally, our growth assays demonstrating that increasing expression of ubiquitin in *dfm1Δ*+Hmg2 cells restores normal levels of growth, indicating that the reduction in free ubiquitin is at least partially responsible for the stress in *dfm1Δ*+Hmg2 cells. Further, we have added Supplemental Figure 6A-B (shown below) showing monomeric ubiquitin in relevant knockout strains without expression Hmg2 leads to no significant changes in monomeric ubiquitin in WT versus *dfm1Δ* cells.

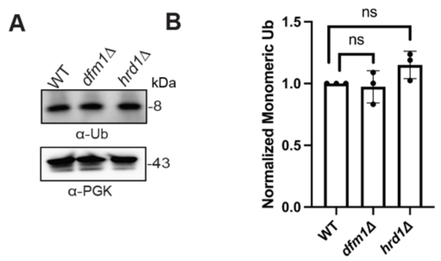

We have now added this section on lines 702-710 of the discussion section to more thoroughly explain the biological relevance of this reduction, “The reduction we observed in monomeric ubiquitin in *dfm1Δ* +Hmg2 cells was approximately half of that observed in WT+Hmg2 cells (Fig. 6E). The hypothesis that this reduction is enough to contribute to toxicity in these cells is supported both by our experiment demonstrating the exogenous ubiquitin restores growth in the

substrate-toxicity assay (Fig. 6D) and by the observation that ataxic Usp14-deficient mice only show about a 25% reduction in monomeric ubiquitin in most tissues (Anderson et al. 2005)."

We have changed instances of saying ubiquitin was depleted to more accurately reflect our finding that ubiquitin was *reduced.*

The fact that many other single KO strains led to the similar phenotype as dfm1 delta strain suggests that this phenomenon might be broader and can be rooted in dfm1-indirect or parallel pathways (as also suggested by the authors at some parts).

We do agree there are likely several parallel pathways that help to contribute to preventing misfolded membrane protein areas and adapting cells to this stress. We believe Dfm1 is one of these pathways, and that Dfm1 functions in a direct manner, but that does not preclude its function in parallel pathways. We have edited the section on the Rpn4, Ubp6, and Dfm1 double knockouts to clarify this (line 610-623):

"Absence of Deubiquitinases and RPN4 in Combination with DFM1 do not Exacerbate Toxicity

We tested double knockouts of *dfm1Δrpn4Δ, dfm1Δ ubp6 Δ,* and *rpn4 Δubp6Δ* cells expressing Hmg2 in the substrate-toxicity assay to determine whether these genetic backgrounds display the same or different growth defect than either of the single knockouts. Expression of either Hmg2 or Ste6* in either *dfm1Δrpn4Δ* or *dfm1Δubp6Δ* cells resulted in a growth defect that phenocopied that observed in any of the single knockouts (Fig. S7A&B), whereas expression of CPY* showed no growth defect (Fig. S7C). In contrast, *rpn4Δubp6Δ* cells showed a growth defect in the absence of substrates whereas *rpn4Δ* and *ubp6Δ* displayed normal growth. Moreover, *rpn4Δubp6Δ* cells along with expression of Hmg2 or Ste6* resulted in synthetic lethality (Fig. S7A-C). This indicates that there is an exacerbation of stress in *rpn4Δubp6Δ* background, whereas there is no increase in toxicity when RPN4 or UBP6 are knocked out in combination with DFM1. It is likely that there are several parallel pathways contributing to preventing stress from misfolded membrane proteins and resolving this stress, and Dfm1 appears to be one of the major mediators of misfolded membrane stress prevention.

Moreover, in all bar figures, it is not clear how the significance of the t-test was defined (what p-value cutoff was used, what are the parameters of t-test were used). Seems that some of the changes are not statistically significant (6B 5h induction, 7E).

Thank you for pointing this out. We apologize for not being clear with the statistical analyses used. The following lines have been added to the figure legend for Figure 6, "Data information: For (B) and (C), all data are mean ± SEM, with 7 biological replicates (N=7). For (D), (E), and (F), all data are mean ± SEM, 3 biological replicates and 2 technical replicates (N=3); statistical significance is displayed as two-tailed unpaired t test, *P<0.05, ns, not significant.

The following lines have been added to the figure legend for Figure 7, "Data information: All dilution growth assays were performed in 3 biological replicates and 2 technical replicates

(N=3). For (F), all data are mean ± SEM, 3 biological replicates (N=3); statistical significance is displayed as two-tailed unpaired t test, *P<0.05, ns, not significant. Solubility assay in (H) was performed with 3 biological replicates (N=3).”

In addition, Fig 2A was already published in Cell Reports and was also used here. This figure should be modified and additional labeling of the DFM1 motifs should be added.
We have updated the schematic in Figure 2A (shown below) and it now clearly labels/lists all of Dfm1 motifs/mutants used in this study. This is much clearer than the previous schematic. Thank you!

A

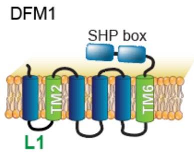

Dfm1 region	mutation	function impacted
L1: WR motif	W63A/R64A	substrate binding
L1	F58S	substrate binding
L1	L64V	substrate binding
L1	K67E	substrate binding
TM2	Q101R/F107S	lipid thinning
TM6:Gx3G motif	G206A/G210A	structural integrity

To be useful for the community, the authors should address the following concerns.
Major comments:

1. Chaperone-activity of dfm1 (line 228). The paper does not show any direct chaperone activity of DFM1, thus, this phrase and the related statements are confusing. The aggregation clearance effect might be direct or indirect. This should be discussed and the term "chaperone" should not be used in this context.

We have now changed all instances of chaperone, in the context of Dfm1, to “chaperone-like.” We do believe that this “chaperone-like” function is a direct effect of Dfm1. Our reasoning is fleshed out in the discussion section (line 635-643) where it states that, “Our results from both this study and previous work from the lab on Dfm1’s function indicate Dfm1 acts directly on misfolded membrane proteins to promote their solubility (Nejatfard et al. 2021). Firstly, all of the L1 mutants of Dfm1, which have previously been shown to ablate binding of Dfm1 to misfolded membrane proteins, such as Hmg2, are not able to promote solubility of Hmg2. Secondly, we demonstrate here that solubilized Hmg2 in DDM still interacts with Dfm1, as shown through co-immunoprecipitation (Fig. 3C). Lastly, both human Derlin-1 and Derlin-2 are able to restore solubility of Hmg2 in *dfm1Δ* cells (Fig. 4F). It seems unlikely that if Dfm1 was influencing solubility of Hmg2 through an indirect route that mammalian derlins that have diverged significantly from Dfm1 would still influence solubility.”

We have additionally added new co-IP data to Figure 3C showing that Dfm1 interacts directly with solubilized Hmg2 (shown below).

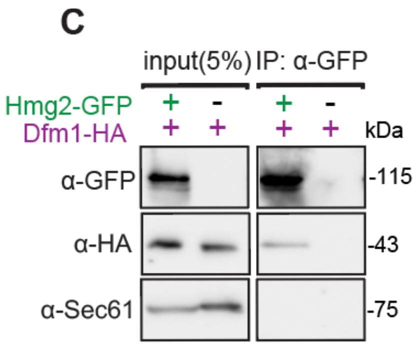

Characterization of the anti-aggregation activity of DFM1:

Fig 3 A-B and 4B are one of the most important figures in this manuscript, however, no statistics was shown.

Thank you for your suggestion. In these experiments, we see an all or nothing effect on aggregation. Because of this, we believe these results are more qualitative than quantitative and do not think statistics is needed for these experiments.

Moreover, any idea why there are large differences between "total" and "pellet" or "sup" fractions, especially in Fig 4 D? Alternatively, Fig. 4C and E suggest that there is the degradation of the tested proteins (high level in T and a small level of proteins in S). Also, differences in levels of Hmg2 in 4A might point to this.

Thank you for pointing this out. Our total fraction is total protein, while pellet and sup are both derived from ER microsomes. In this protocol, we don't have enough samples to collect total ER fraction. There are a few potential sources for these differences, both experimental limitation and biological differences. First, because the total is collected further upstream in the protocol than pellet or soluble, it is likely that some protein is lost as we go through the protocol. In previous work from Dr. Neal and Dr. Hampton, soluble Hmg2 was more prone to stick to the plastic microcentrifuge tubes (due to hydrophobic residues being exposed on Hmg2) if the tubes were not pre-coated with BSA, so this is a potential source where Hmg2 is not fully recovered (Neal, Bennett, and Hampton 2017). Additionally, a scenario where there is less total compared to pellet or supernatant appears to be specific for Ste6* (Fig. 4D). Ste6* is known to be targeted by the cytosolic E3 ligase Ubr1 (Stolz et al. 2013), so it seems likely that there was some degradation of the protein in the total fraction that could account for this discrepancy. Regardless, the soluble versus aggregated protein is all or nothing for all misfolded membrane proteins tested in dfm1 null strains.

The authors tried to detect aggregates in cells using confocal microscopy, however, this analysis was not very successful as also mentioned by the authors (S1). The provided images in S1 are very strange. It is unclear where are the bounders of the cells, seems like these are dead cells or may be vacuoles. GFP-Hmg2 was successfully used by others, for example in Schafer et al.,

EMBO 2019, Hampton et al., PNAS 1996. An improvement of these assays in this study can be very useful.

We would not characterize this analysis as unsuccessful, but rather as negative data that demonstrated these Hmg2 aggregates cannot be identified through confocal microscopy. We have updated the images to show a wider field of view (Figure S1D, shown below), where the cells are not dead.

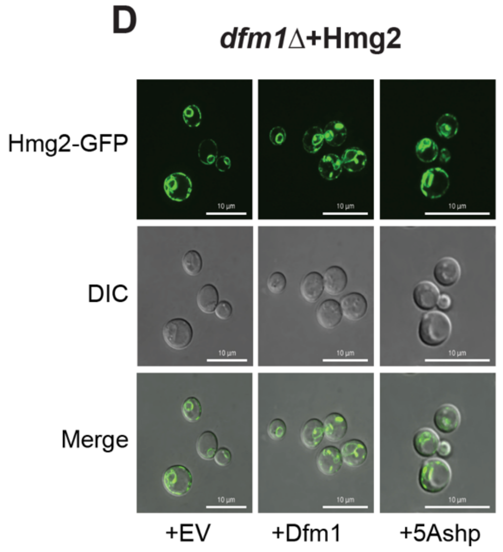

2. The authors showed very nicely that different substrates, Hmg2, Pdr5*, Ste6*, CFTR variants undergo aggregation in the dfm1 delta strains, and that it does not depend on the Cdc48 binding domain. The authors also tested levels of free ubiquitin in the cells expressing Hmg2 and detected a slight decrease in the dfm1 cells relative to hrd1 delta strains (not clear if it is really statistically significant due to large errors). Together with phenotypic studies that show growth defects in hrd and doa delta strains, the authors propose that "membrane substrates are ubiquitinated in delta dfm1 strains but not in delta hrd1, doa1" (line 132) - there is no experimental support for this in this paper.

Thank you for pointing out this experiment. We have updated the paper to include blots for Hmg2 and Ste6* that were immunoprecipitated from various yeast strains and then blotted for ubiquitin. This is now Figure 7B (shown below).

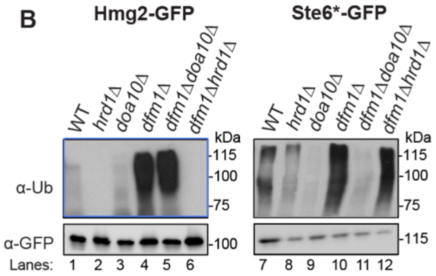

Later on, the authors show that the same growth defect is common to dfm1 delta, rpn4 delta and pdr1 delta strains. These can be due to related or non-related pathways.

Pdr1 null cells does not show a growth defect, despite having large target overlap with Rpn4. As discussed earlier in this rebuttal, we edited the results section that now thoroughly discusses the Rpn4 Dfm1 double knockout to clarify that they could be acting in parallel pathways. Thank you.

2. Lines 178 - 184 . Are the expression levels of the Dfm1 variants similar to the wt?

Thank you for pointing this out. We have added western blots showing expression levels of WT and mutant Dfm1 in the strains used in the growth assay to Supplemental Figure 1C (shown below).

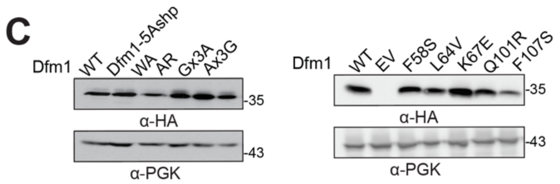

4. lines 196-204 - Fig 6G is incorrect. I cannot find the related figure to this statement. Moreover, the relevance of this part is not very clear or developed.

Our apologies, this was a typo and should have read Figure 2G. This has now been updated. In addition, we have added Figure 4F (shown below), which show human derlins, Derlin-1 and Derlin-2 can also promote solubility of Hmg2 when expressed in yeast cells.

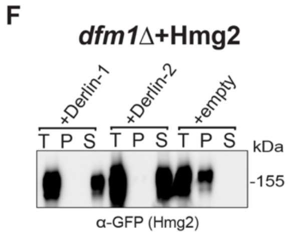

5. lines 263-270- Fig 2A-H does not relate to this sentence. No time dependence was shown in any figure.
The statement "Unexpectedly, the level of UPR activation was much higher in dfm1Δ cells expressing CPY* than in pdr5Δ cells" is not supported by the S2E-F figures. It looks the opposite. The background in delta dfm1-CPY* is high with or without induction of CPY*. Maybe it is true for shorter incubation time? But it is not shown in the provided figures.

Thank you for pointing out this error. You are correct that there was a trend with earlier time points, by then a drop off by 5 hours, possibly due to cell death. This analysis was in an earlier

draft of the paper but was removed and we did not follow up to confirm cell death was occurring. We have removed this portion of the results section.

Line 276 " increases sensitivity to other stresses" - it is not tested here, so it is very speculative at this point.

This line has been removed. Thank you.

6. Transcriptomic analysis of dfm1 delta cells expressing Hmg2. I am not sure this part adds to the story, I found it very confusing and not developed. I think it can be removed and used in another manuscript.

We also agree and have moved this data to supplemental Figure 3 and moved the UPR analysis to Figure 5 instead.

There is inconsistency in the used strains: in the results and methods session- dfm1 delta+ Hmg2 cells but in the figure - double mutant, dfm1 and pdr5 delta. If indeed the analysis was done in the double mutant strain then it is not relevant to results discussed previously and additional controls should be added (growth phenotype, solubility test and others).

These *dfm1Δ* cells were acquired from a yeast knockout collection, which is a slightly different strain background than the lab background we generally use (BY4742 versus S288C). Because there is no trule "wildtype" strain background for knockout collection, *pdr5Δ* cells are commonly used as the WT strain. Additionally, because Pdr5 is a drug pump, it is essential to knock it out for experiments where cells are treated with drugs, like MG132. We acknowledge that this is very niche knowledge that is probably confusing to those that don't work with *S. cerevisiae*, or even just with this background. This is compounded by the fact that we also use Pdr5*, a misfolded ERAD-targeted variant of Pdr5, at several points in this study. We have added the following sentence to line 355 as well as a growth assay showing that these strains have a growth defect to supplemental figure 3A (shown below), "These yeast strains were generated from a yeast knockout collection with the BY4742 strain background, and *pdr5Δ* cells are commonly used as the wildtype background for the knockout collection. We validated using the substrate-toxicity assay that *dfm1Δ pdr5Δ* +Hmg2 strains in this background also display a growth defect (Fig. S3A)."

A

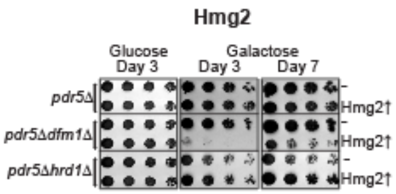

Moreover, an additional table should be used to support the overlap between Rpn4 targets and the identified dfm1-related genes (lines 318- 319)

This table has been added to Supplemental Figure 3C.

Genes targeted by Rpn4 targets			
BDF2	PUP3	BMH1	RPN12
UBI4	PRE4	RPT6	
NCA3	PRE10	RPT1	
PRE1	RPT2	PRE2	
CDC48	PRE8	PUP2	
PRE3	SCL1	PUP1	
PRE9	PRE6	RPT5	

7. Fig 7A : the phenotype in dfm1 delta strain is less strong than in Fig 1. Thus, to compare different strains, consider conducting a complementary test, such as growth in medium or others. In addition, Fig 7A cannot be a basis for the statement in line 387: "This indicates that growth stress in dfm1Δ cells is dependent upon ubiquitination of the accumulated misfolded membrane protein." No ubiquitination analysis was provided here.

It seems there was an issue with this particular growth assay in Figure 7A where the strains in one of the replicates had started to suppress the retrotranslocation defect (a phenomenon we have thoroughly investigated in Neal et al., iScience, 2020), or perhaps there was an issue with the galactose plates that did not result in strong expression of substrates. Regardless, we have replace the imaged with one of cells that display a much more prominent effect and performed another replicate of the experiment to ensure that the mild phenotype was not a persistent issue.
The updated panel for Figure 7A is shown below.

In respect to ubiquitination state of substrates in these strains, we have added new data to Figure 7B that shows ubiquitination of immunoprecipitated Hmg2-GFP and Ste6*-GFP in the double knockouts.

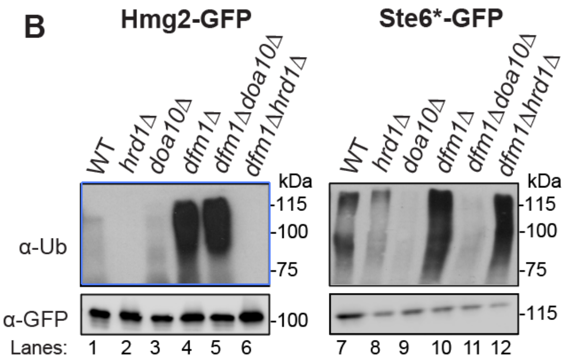

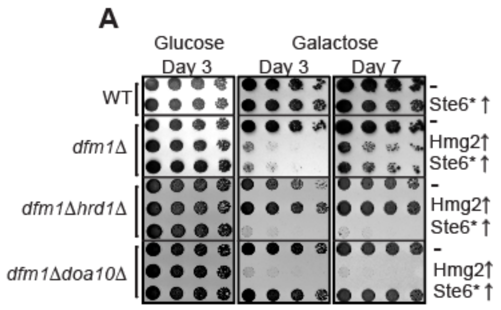

Fig 7B - it is still not clear to me why the expression of the Hmg2 double mutant K6R-K357R has such a strong recovery effect (similar to wt growth) while single mutants lead to significant cell death.

The double mutant would be expected to phenocopy the K6R mutant in the growth assay, as inclusion of the K6R mutant means that Hmg2 is completely stabilized. We have changed the sentence starting on line 570 to clarify this point, “Moreover, the growth defect is still observed in the double mutant Hmg2- (K6R, K357R), which phenocopies Hmg2-K6R, in that it is completely stabilized, consistent with the model that growth stress in the absence of Dfm1 is dependent on the accumulation of ubiquitinated membrane substrates (Fig. 7C).”

Moreover, the quality of figure S8 is not very good. Is it possible to use CFU values?
We will ensure in resubmission that the image quality remains higher for this figure.

Minor comments:

1. Fig 2A. low resolution, very difficult to distinguish between different motifs colored by very close colors. The figure legend does not correspond to the colors used in the figure. Should be modified and the motifs should be labeled better. Moreover, this figure is already used in another publication.
In addition, motif GxxxG (line 175) is not labeled in the figure. Shp1 domain should be shown in 2A.

A new schematic (shown below) is now used in Figure 2A.

A

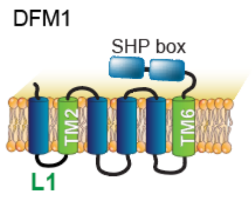

Dfm1 region	mutation	function impacted
L1: WR motif	W63A/R64A	substrate binding
L1	F58S	substrate binding
L1	L64V	substrate binding
L1	K67E	substrate binding
TM2	Q101R/F107S	lipid thinning
TM6:Gx3G motif	G206A/G210A	structural integrity

2. Fig 2: there is no consistency between the B, C and E panels in the terms of order (+DFM1 followed by -DFM1).

We have updated the strain order for Figure 2C for more consistency. Thank you.

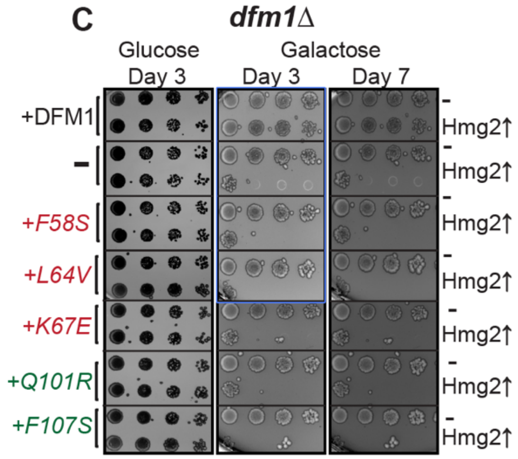

3. line 260 - what does "optical reporter" mean? Consider rephrasing
Optical reporter is not uncommon as a phrase used for reporters that utilize fluorescence as a readout. We have altered this to just say reporter.

4. Fig 5. Panel B is after D
This has been updated and this figure is now supplemental figure 3.

5. Fig 5 - what are the p-values (and what test) were used for the functional enrichment?

We did not use p-values for technical enrichment. The PCA analysis was done by Dr. Sascha Duttke and Dr. Chris Benner (co-authors on this paper), who are both experts in RNA-seq and are both bioinformaticians who have created new sequencing techniques and new data processing and analysis pipelines. Both agreed this was the best way to analyze the data.

Generally, analysis of RNA-seq with programs like Deseq2 will only compare one sample versus one other. Because we had six different strains and wanted to know what was uniquely different between *dfm1Δ pdr5Δ* +Hmg2 strains versus all other strains tested, we opted for a principal component analysis to find which genes had the most similar expression levels in both of the *dfm1Δ pdr5Δ* +Hmg2 replicates versus all other strains and then compared this list to fold changes. The reason we don't see massive changes in expression of any one gene is that we were looking at changes that occurred within a few hours of inducing Hmg2 expression. Essentially, because the number of newly produced RNAs is low compared to the overall pool of RNAs, there are no massive changes in gene expression. We did attempt to use the Click-iT Nascent RNA Capture Kit to resolve this issue and show higher enrichment levels, but we were unable to successfully produce libraries with this kit.

6. line 334 . Pre6-GFP - should be explained what Pre6 is and why it was used here.

Thank you for pointing this out. Line 418-420 now reads, "Pre6 is a component of the 20S core of the proteasome that can be transcriptionally upregulated by Rpn4 and Pre6-GFP has been used by others as a marker for the proteasome (Enenkel, Lehmann, and Kloetzel 1998; Xie and Varshavsky 2001).

7. Fig 6B and C - details about the t-test analysis are needed. Doesn't look very significant (pink and green bars). 6D-F : dfm1 delta should be shown as well.
The colors are very confusing because of the previous panels. Consider do not use colors in the SUS-GFP bars as this is a different experiment and can be confused with the background of cells (expressing Hmg2, CPY* etc).

The following lines have been added to the figure legend for Figure 6, "Data information: For (B) and (C), all data are mean ± SEM, with 7 biological replicates (N=7). For (D), (E), and (F), all data are mean ± SEM, 3 biological replicates and 2 technical replicates (N=3); statistical significance is displayed as two-tailed unpaired t test, *P<0.05, ns, not significant.

Thank you for the suggestion on color for 6D-F, we have changed this to different solid colors. Dfm1 delta is not shown because for the MG132 sensitivity because the drug pump Pdr5 must be knocked out to allow MG132 to be effective in cells. We opted to test a *dfm1Δ hrd1Δ pdr5Δ*+SUS-GFP versus a *dfm1Δpdr5Δ*+SUS-GFP because we used a consitiutively expressed substrate, which causes *dfm1Δ* cells to rapidly suppress their retrotranslocation defect (Neal et al. 2020) and we wanted to avoid false positive colonies in this assay.
